# Determining Key Factors for the Open-Loop Control of Molecular Fragmentation Using Shaped Strong Fields

**DOI:** 10.1021/acs.jpclett.4c02889

**Published:** 2024-12-12

**Authors:** Jacob Stamm, Sung Kwon, Marcos Dantus

**Affiliations:** † Department of Chemistry, Michigan State University, East Lansing, Michigan 48824, United States; ‡ Department of Physics and Astronomy, Michigan State University, East Lansing, Michigan 48824, United States; § Department of Electric and Computer Engineering, Michigan State University, East Lansing, Michigan 48824, United States

## Abstract

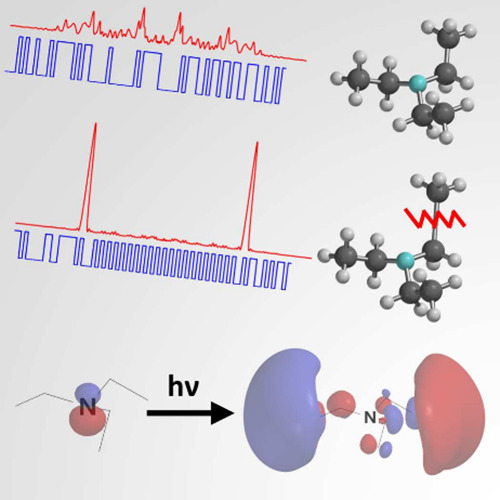

Pulse shaping has long been employed for tailoring femtosecond laser pulses to study and control the fragmentation of polyatomic molecules. In many cases, a physical explanation connecting the properties of the field to the observed control is difficult to ascertain. We utilized 80 bit binary spectral phase functions to parametrize and map the search space, gaining insight into which pulse parameters most impact the ion yield and fragmentation pattern for the relatively large triethylamine [N(C_2_H_5_)_3_] molecule. Pulse structures used to control the *m*/*z* 86 branching ratio beyond a simple intensity dependence are identified and compared to pump–probe results. All of these findings are explained in terms of control via a dissociative Rydberg state in the neutral molecule. This methodology may be used to discover new control mechanisms and shed light onto which pulse parameters most influence the interaction between strong field lasers and matter.

Shaped femtosecond laser pulses have been widely used to study and control chemistry.^1−3^ Several schemes to control the results of the light–matter interaction have been proposed over the years, with one of the most modern and versatile of them being spectral phase manipulation.^4−10^ This important control parameter, describing the relative phase of the different frequency components making up the ultrashort pulse, allows for customization of the temporal electric field profile. With the number of possible spectral phase functions (and thus the number of custom laser pulses) exceeding 10^100^, some guiding principles are required to determine and predict how such a customized laser pulse will interact with a given system. One approach to gain insight into these principles is an open-loop search experiment, which involves evaluating an entire space or subspace of pulse shapes and assessing their control over a specific outcome for each individual pulse.^11^ This approach has the advantage of covering a representative range of pulse shapes with predefined parameters, some of which may be chosen based on theoretical modeling or tailored to target specific influences on the system under study.^10^

The problem with open-loop experiments is, to control more complicated chemical phenomena, the pulse may require a richer structure that may not be entirely represented by the parametrization of the space of phase functions the experimenter chose. To evaluate all possible spectral phase functions in an open-loop manner would require the evaluation of a large number of phases, with the number being equal to the phase resolution elevated to the number of pixels in the pulse shaper (which is 4096^800^ ≈ 10^2888^ for the pulse shaper used here). One method to circumvent this limitation is to use binary pulse shaping (BPS), which significantly reduces the space of all spectral phase functions by using only 0 and π phase values, reducing the space to 2^800^ possible masks. Further dimensionality reduction can be achieved by grouping adjacent pixels into blocks and allowing the set of blocks to take only 0 and π phase values, making a systematic or representative search more feasible.^11−14^ The benefit of BPS is that it can approximate any spectral phase function (Figure 1) while reducing the dimensionality of the phase space that needs to be searched. This retains the capability of uncovering various control mechanisms while allowing for a variable-sized search space (via the number of bits) that may be scaled depending upon the number of desired parameters and the system to be optimized.^15^

**Figure 1:**
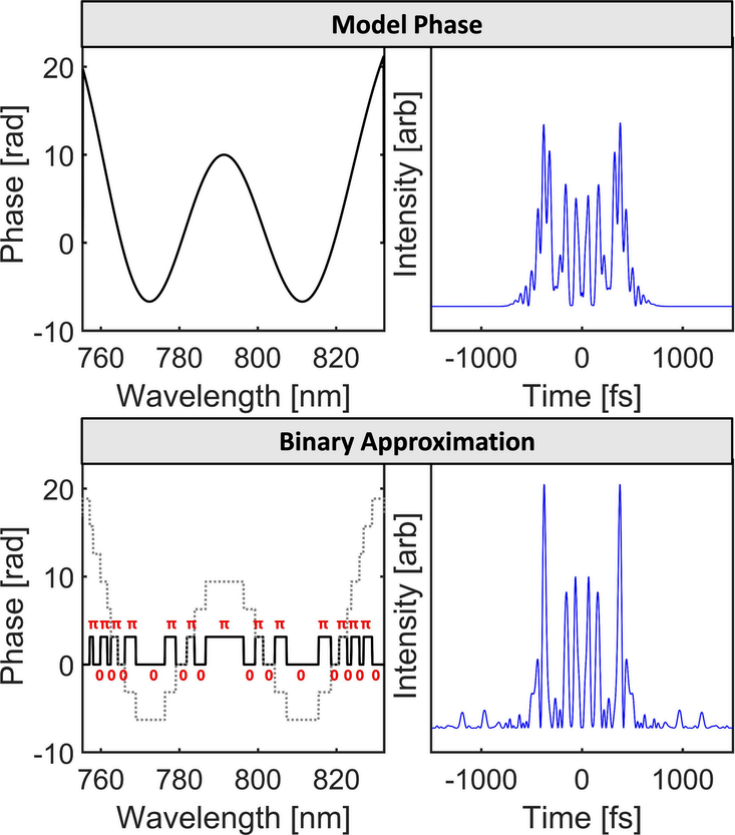
Binary approximation of a spectral phase function. A model phase (top left) and its calculated temporal profile (top right) are approximated using only 0 and π phases (bottom panels). The 80 bit binary phase can be unwrapped (dotted line) to show the approximation of the model phase (bottom left panel).

Note that other parametrizations besides BPS are possible for open-loop searches and could be applied in an open-loop manner using the same methodology outlined here. Some of these alternatives include sinusoidal phase control, which is particularly effective for generating pulse trains,^16−18^ and polynomial expansions, which are practical for pulse compression as pulse broadening is primarily influenced by low-order components (second to fifth).^19^ However, representing any arbitrary phase function using these parametrizations would necessitate a large sum of sinusoidal functions (as in a sinusoidal transform) or high-order polynomials (as in a Taylor expansion). Dependent upon the optimal phase function, a large number of parameters may be required in these expansions. Here, BPS was chosen due to its simplicity and reproducibility^20^ (only 0 and π phase values need to be calibrated) as well as its tailoring toward controlling quantum excitation pathways through constructive (0-phase difference) or destructive (π-phase difference) pathway interference.^21^

One area where phase shaping can be applied to control chemical outcomes is the fragmentation of large polyatomic molecules. Controlling molecular fragmentation poses a significant challenge due to the large number of degrees of freedom and potentially complex, interconnected fragmentation channels. Nonetheless, these control experiments may offer the best possibility for improving real-world applications, such as isomer identification.^22^ Here, we search for pulse parameters that control both the ionization and fragmentation of triethylamine [N(C_2_H_5_)_3_] in an open-loop manner using binary phase shaping. While control experiments typically seek to explain why a specific phase function achieves a desired product ion yield, we instead search for general explanatory variables that can be used to predict the performance of any phase function. It is expected that using an open-loop search with BPS parametrization will enable the extraction of critical pulse parameters necessary for controlling molecular fragmentation. This approach may reveal new control mechanisms without *a priori* knowledge of the system under study.

Details of the experimental methodology are presented in the Experimental Methods of the Supporting Information. Briefly, 795 nm, 35 fs laser pulses were shaped using a calibrated pulse shaper and focused into a time-of-flight mass spectrometer. Triethylamine vapor was leaked into the mass spectrometer through a needle valve, and its resulting mass spectrum following ionization by the laser was recorded for multiple series of binary phase-shaped pulses. The mass spectrum of triethylamine under several excitation conditions is shown in Figure 2. The triethylamine cation (*m*/*z* 101) dissociates into several fragments, with the heaviest being *m*/*z* 86 (methyl loss) and the subsequent lowest energy pathway being the fragmentation of *m*/*z* 86 into *m*/*z* 58 (methyl + ethylene loss) and then into *m*/*z* 30 (methyl + double ethylene loss).^23^ Additionally, for pulses with a higher peak intensity, the fragments *m*/*z* 42 and 44 (double ethylene loss with hydrogen migration), *m*/*z* 12–15 (CH_*n*_^+^, where *n* = 0, 1, 2, and 3), and *m*/*z* 1 (H^+^) appear. The transform limited (TL) pulse is well within the saturation regime, but the decreased peak intensity of the 80 bit BPS pulses put them below this threshold and well within in the regime where coherent mechanisms play an important role (Figure S1 of the Supporting Information).

**Figure 2:**
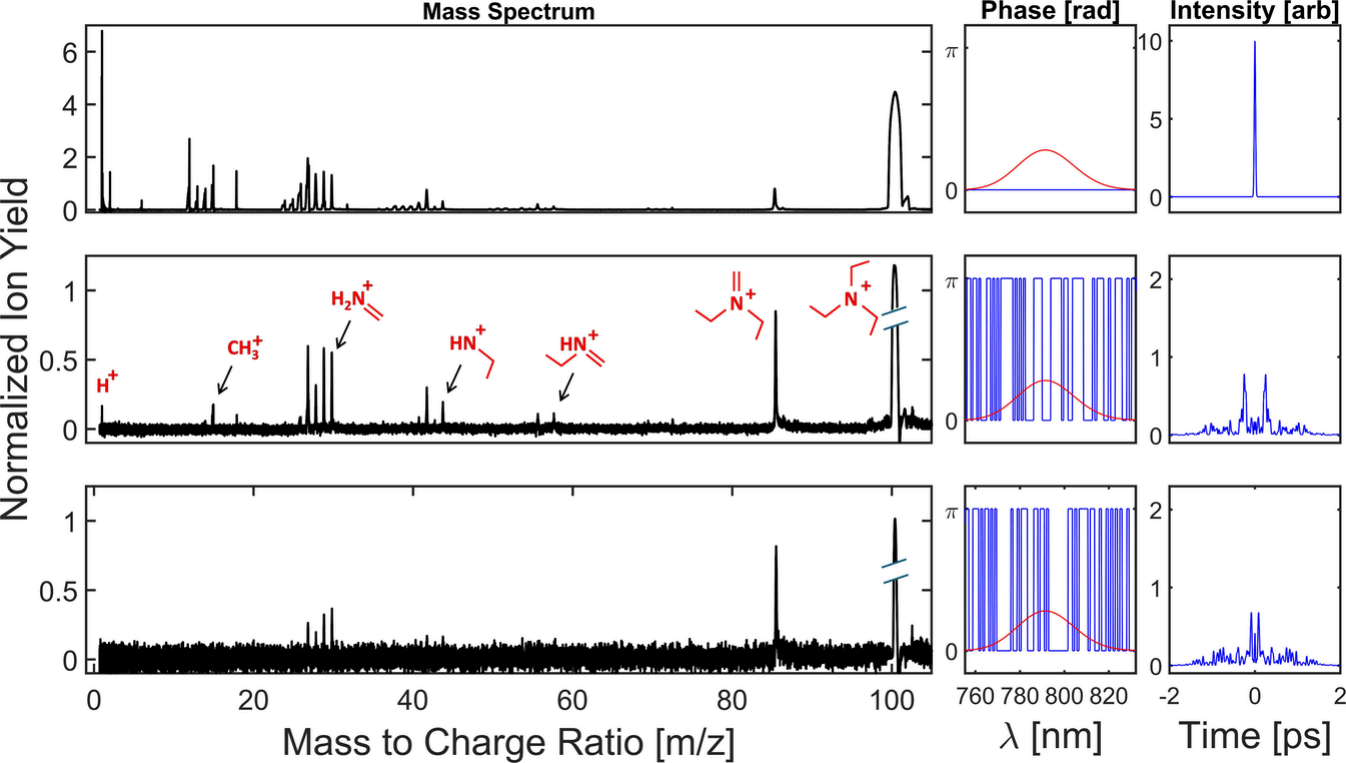
Mass spectrum of triethylamine (first column) following ionization with a transform limited pulse (first row) and two different binary phase-shaped strong-field pulses (second and third rows). All spectra have been normalized to the total integrated ion signal. In the second row, key peaks are labeled in red with probable structures. For visibility, the molecular ion has been scaled down by a factor of 1, 10, and 20, respectively. The spectral phases (second column, blue) for each pulse only take values of 0 and π across the pulse’s spectrum, which is shown in red. The calculated temporal intensity is shown in the third column. The intensity of the single transform limited pulse used to ionize was ∼3 × 10^14^ W cm^–2^.

The goal of the present investigation is to search the space of spectral phase functions and extract how the pulse parameters affect both the total ion yield and the branching ratios within the fragmentation pattern of triethylamine. As mentioned above, BPS with 0 and π phases was used to reduce the size of the phase space at the expense of lower phase resolution and symmetrized pulses. Two examples of 80 bit random binary phases are shown in the right panels of Figure 2.

To correlate the calculated pulse parameters with both the total ion yield and fragmentation pattern, 3200 unique 80 bit binary phases were used to ionize and fragment triethylamine. Specific details about the laser pulse parameters, phase shaping, ion detection, and averaging conditions are contained in the Experimental Methods of the Supporting Information. We will focus on controlling the branching ratio of one of the main fragment ions of triethylamine, *m*/*z* 86, which corresponds to the molecular ion with one methyl loss. To determine the effect of BPS on the branching ratio of *m*/*z* 86, its yield was normalized to the total ion yield and tracked for the sample set of 3200 BPS masks (Figure 3). The intensity of the ionizing pulse is a well-known parameter that impacts the fragmentation pattern of molecules and must be accounted for to search for non-trivial control schemes (Figure S2 of the Supporting Information). This is accounted for by tracking the normalized *m*/*z* 86 yield relative to the integrated second harmonic power spectrum (*I*_SHG_), which is closely connected to the peak electric field strength.^8,24^ From Figure 3, the normalized yield of the *m*/*z* 86 ion is generally anticorrelated with *I*_SHG_. This is to be expected, because *m*/*z* 86 is one of the first product ions to be formed when increasing the internal energy of triethylamine (8.4 eV appearance energy compared to 7.5 eV ionization potential of triethylamine^25,26^), so increasing the intensity of the pulse decreases the *m*/*z* 86 branching ratio by opening up other fragmentation channels to compete. However, *I*_SHG_ does not appear to be a single parameter explanation for the yield of *m*/*z* 86. With the confounding intensity parameter (*I*_SHG_) taken into account, the remaining variance in the *m*/*z* 86 branching ratio is a sign of non-trivial control. There are BPS masks that produce 2× more *m*/*z* 86 for a given *I*_SHG_ value, a disparity that cannot be accounted for due to noisy measurements (see the black noise level line in Figure 3). Understanding the pulse structures that explain this yield variance beyond the noise floor is the essence of our use of open-loop random searches of the phase space to determine coherent control schemes.

**Figure 3:**
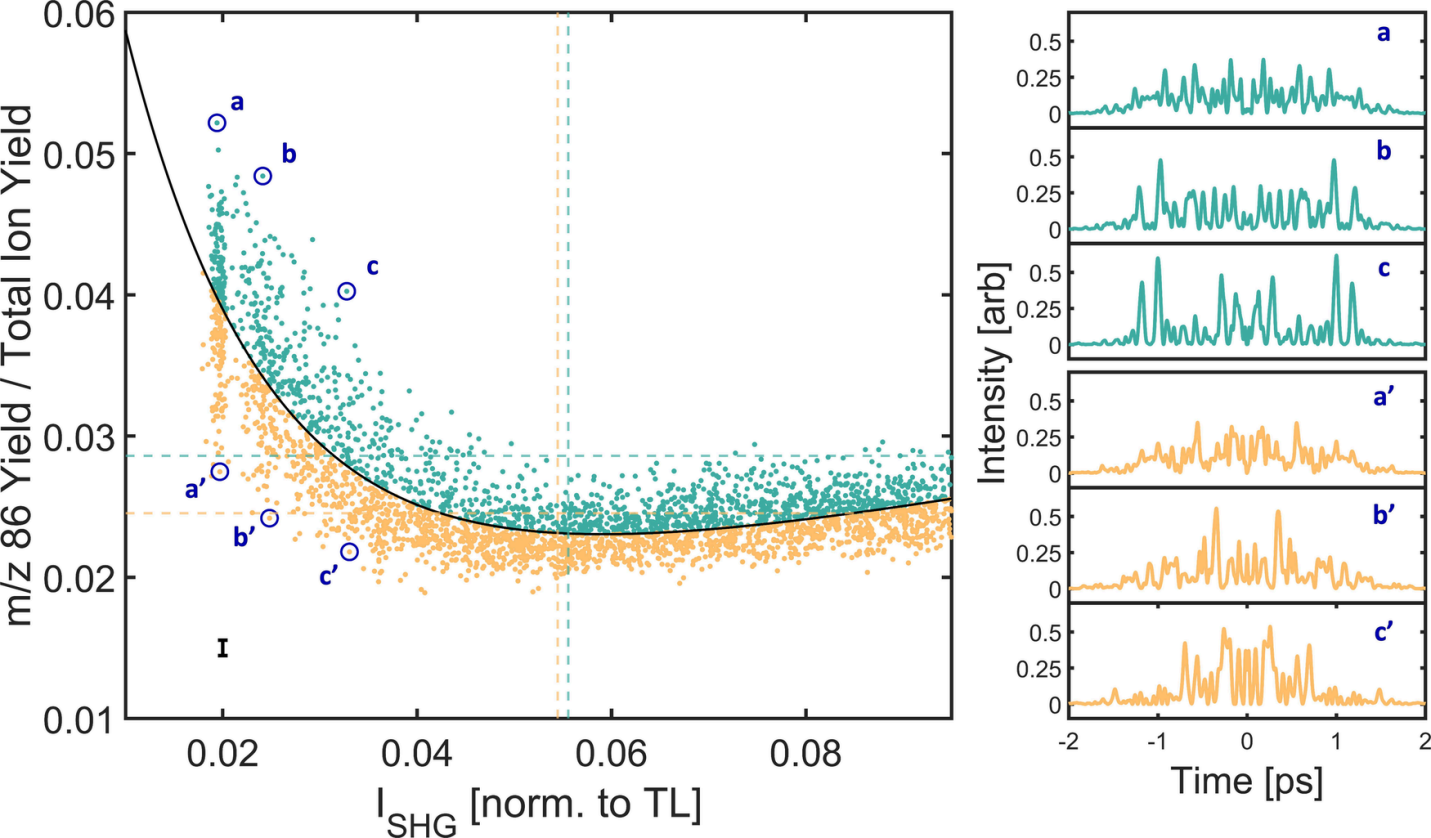
Yield of the *m*/*z* 86 ion (normalized to total ion yield) versus integrated second harmonic intensity (*I*_SHG_) following the irradiation of triethylamine with 3200 unique 80 bit binary-shaped pulses. The data are fit to a biexponential function (black line), and phases above this line are colored turquoise, while phases below this line are colored tan. The average normalized *m*/*z* 86 yield and *I*_SHG_ values for both of these groups are shown as dashed lines. The black line in the bottom left of the scatter plot indicates the ±1σ noise level. The temporal profiles of three selected pairs of masks (a/a′, b/b′, and c/c′, blue circles), each with similar *I*_SHG_ values but large differences in the *m*/*z* 86 yield, are plotted in the right panels.

To investigate what causes the variation in the *m*/*z* 86 yield between masks of similar *I*_SHG_ values, several pulse parameters for temporal envelopes above and below the fit line in Figure 3 were calculated (Figure S3 of the Supporting Information), such as the peak field strength, spread in the temporal profile, number of “switches” in the binary phase mask, instantaneous frequency, integrated harmonic spectra up to the fifth order, pulse trains in the temporal profile, and temporal partial autocorrelation function (PACF). No explanation for the 2× difference in the normalized *m*/*z* 86 ion yield for masks of similar *I*_SHG_ values was found, with the exception of the PACFs. These PACFs are simply the correlation of a temporal profile with a delayed copy of itself and are calculated for a series of time delays of the copied profile (generally referred to as lags). This value is proportional to
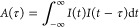
1where *I*(*t*) is the temporal intensity profile of a given pulse, which is calculated via the Fourier transform
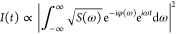
2where *S*(ω) is the pulse spectrum and φ(ω) is the spectral phase. These PACFs, when calculated as a function of time lag (τ), test if the specific spacing between pulse structures is correlated with the enhancement. The mean PACFs for the two groups as a function of time lag is shown in Figure 4. To calculate these quantities, we first compute the PACF for each temporal profile in both groups (turquoise and tan) as a function of time lag. Next, we average the PACFs of all masks in each group, resulting in two mean PACFs, illustrated in turquoise and tan. The mean autocorrelations between the temporal profiles that make more (turquoise) or less than expected *m*/*z* 86 (tan) have notable differences (black line, top left panel). This is in contrast to other ions, such as *m*/*z* 30 (top right panel), which show no appreciable differences in PACFs of the two groups. Critically, the group that produces disproportionately more *m*/*z* 86 has a larger mean autocorrelation for a time lag of ∼2 ps, meaning that binary phase masks that generate structures separated by ∼2 ps will tend to produce more *m*/*z* 86 than *I*_SHG_ predicts. This is corroborated by pump–probe measurements (bottom panels of Figure 4) that exhibit the same enhanced ion yield at pump–probe delays of ∼2 ps for *m*/*z* 86.

**Figure 4:**
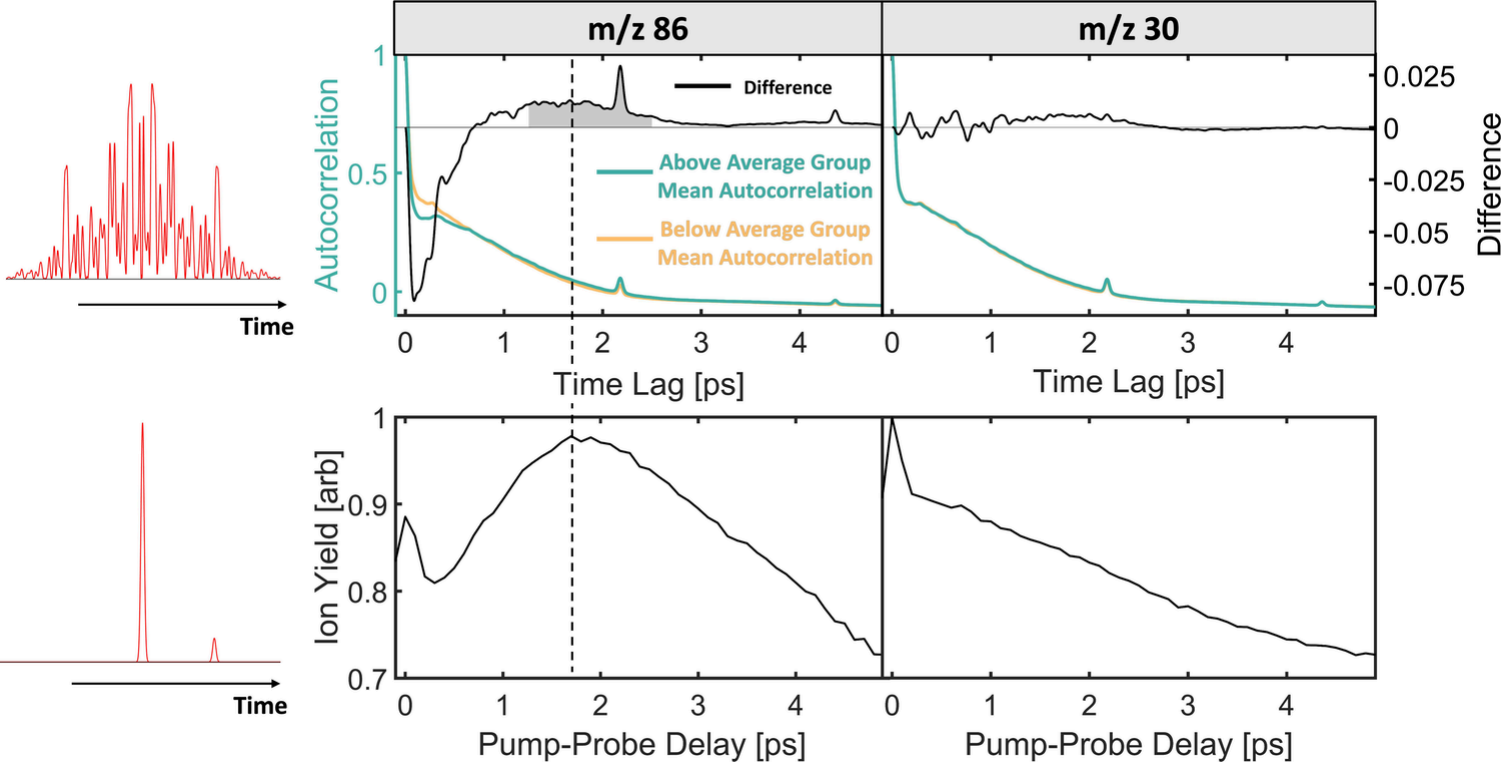
Mean autocorrelations of the temporal intensity profiles that generate more (turquoise) or less (tan) *m*/*z* 86 and 30 than predicted by *I*_SHG_. The difference between these mean autocorrelations is shown as a black line (top panels). Note that there are spikes at multiples of ∼2.2 ps in the autocorrelations that relate to the periodicity of the phase when using equally spaced 80 bit phase masks. The normalized yield of *m*/*z* 86 and 30 for a pump–probe scan with identical power is shown in the bottom two panels to illustrate the similarities in dynamics. The gray shaded region shows a selected range of delays (see the text), and the black dotted line is a visual aid to draw a connection between the enhancement seen in the autocorrelations and the pump–probe delay curve.

The agreement with the pump–probe results points to a two-pulse-interaction control mechanism for *m*/*z* 86 generation. We investigated this further by generating another sample set of 80 bit binary masks but only keeping those which have pulse structures separated by 1.25–2.5 ps (gray shaded region in Figure 4). To ensure control beyond trivial parameters, these 1.25–2.5 ps results are compared to randomly generated BPS masks that have identical *I*_SHG_ values. This comparison is shown in Figure 5, where the autocorrelation-selected pulses outperform the randomly generated pulses with the same *I*_SHG_ by about 30%, pointing to control beyond the peak field strength. Additionally, the selected shaped pulses with a double-pulse structure and picosecond spacing outperform both transform-limited pulses (black dashed line) and pump–probe results (red dashed line). This demonstrates an extra enhancement of the BPS-shaped pulses, which might be attributed to low-intensity satellite pulses that are generated at regular periods from the main pulse(s). These satellites are created due to the repetitive phase features in the 80 bit masks and could contribute to neutral excitation while avoiding ionization due to their low intensity. These results, in combination with the TL–TL pump–probe data (Figure 4), suggest that a two-pulse-interaction mechanism is responsible for the control of the *m*/*z* 86 yield in triethylamine.

**Figure 5:**
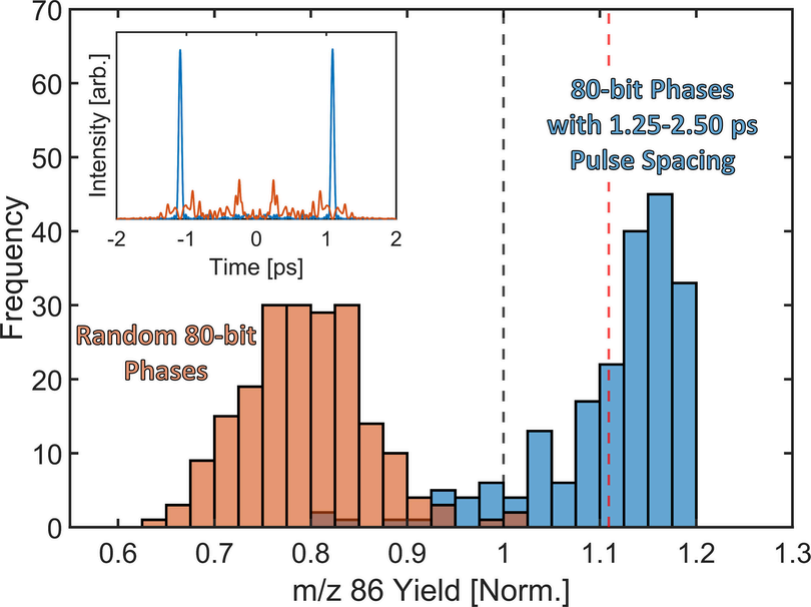
Histogram of the normalized *m*/*z* 86 yield for 200 randomly generated 80 bit binary phases (orange) and 200 generated 80 bit binary phases with pulse structures separated by 1.25–2.5 ps (blue). Both groups have the same integrated second harmonic intensity and have been normalized to the *m*/*z* 86 yield for a single transform limited pulse (black dotted line). The maximum normalized *m*/*z* 86 yield obtained via pump–probe measurements using a V-shaped spectral phase function is shown as a red dotted line. The inset shows the temporal profiles for the best mask in the BPS-spaced pulse group (blue) and worst mask in the random BPS group (orange).

We propose the following hypothesis, pending future experimentation, regarding the control mechanism that is being leveraged by the BPS pulses in this specific system to enhance *m*/*z* 86 production. Triethylamine is a model system for other amines, as this class of compounds has similar excited states and absorbs light around ∼200 nm (four photons of our pump central wavelength).^27^ Thus, the fourth harmonic spectrum is a pertinent quantity that may correlate to excitation to this state, which has been shown previously to be a Rydberg state of the neutral molecule.^28,29^ Multiphoton transitions through these excited states have previously been shown to be strongly sensitive to the wavelength.^23,30^ These findings align with the correlation of the total ion signal with a red-shifted fourth harmonic spectrum toward 200 nm (Figure S2 of the Supporting Information). This indicates that absorption to the Rydberg state of neutral triethylamine aids in further ionization. Similar tertiary amines have several possible Rydberg states around 200 nm excitation: a manifold of 3p states as well as a lower-lying 3s state.^31^ Several studies have identified this 3s Rydberg state to be dissociative due to the σ* character that induces α cleavage, which in the case of triethylamine would lead to methyl loss.^32−34^ Time-resolved photoelectron spectroscopy studies on similar amines have shown that the time scale for an initially prepared bound 3p Rydberg state to internally convert into the dissociative 3s state between 1 and 3 picoseconds.^35^ Thus, because 3s is the only state that is dissociative, we attribute the enhanced *m*/*z* 86 yield to the population of this state following internal conversion. Given that the optimum pulses involve a two-pulse temporal profile, we propose that the first pulse populates the 3p Rydberg state of the neutral molecule via four-photon excitation. The 3p Rydberg state then internally converts to the dissociative 3s Rydberg state within ∼ 2 ps, when the time-delayed second pulse ionizes the system, populating a repulsive state in the cation that produces *m*/*z* 86. The *m*/*z* 86 ion signal decreases at longer times as the hot 3s Rydberg state population vibrationally relaxes, undergoes α cleavage, fluoresces, or internally converts back to the ground state. This proposed pathway aligns with the pump–probe data shown in Figure 4, which displays a rise and decay consistent with the population and subsequent depopulation of a state generated by the pump pulse. It should be emphasized that this dynamical model explains the enhancement of *m*/*z* 86 production caused by the second pulse. The pathway for *m*/*z* 86 production with a single short pulse involves direct excitation to a cationic excited state, which also produces *m*/*z* 86. This ionic pathway contributes to a background signal that is not observed at low intensities. Thus, the ionic pathway adds a background yield onto which the second pulse enhancement is superimposed. The two pathways responsible for *m*/*z* 86 production are illustrated in Figure S4 of the Supporting Information.^36^

While this entire process could occur in the ion state,^37^ with the experimental results being a mixture of neutral and ion state dynamics, we find it more probable given the relatively weak excitation conditions produced by most of the 80 bit BPS masks that a majority of the dynamics are occurring in the neutral molecule. This is supported by pump–probe results on the product ions that may result following the fragmentation of *m*/*z* 86, which are (in sequential order): *m*/*z* 58, 30, and 28.^23^ If the dissociation occurred in the ion state, an enhancement of these fragments would be found at ∼2 ps, as the probe provides the additional energy to fragment the *m*/*z* 86 ion. The pump–probe results do not show such a feature. Additionally, the same enhancement structure at ∼2 ps is observed even when the pump is much weaker than the probe, indicating neutral dynamics. It should also be noted that previous studies have observed bond-selective photochemistry by using different wavelengths to promote non-bonding electrons into anti-bonding orbitals in the neutral molecule.^38^

In conclusion, we investigated the control over the ionization and fragmentation of triethylamine using 80 bit binary-phase-shaped pulses. When accounting for *I*_SHG_, an important confounding variable in control studies, we identified a control mechanism to manipulate the fragmentation of triethylamine into *m*/*z* 86. This was identified to be a two-pulse mechanism that is related to the internal conversion into a dissociative Rydberg state of the neutral molecule. The methodologies and key parameters identified here may provide an open-loop route for discovering new coherent control mechanisms and aid in explaining the results of other control experiments.

## Experimental Section

The experimental methods are detailed in the Supporting Information.

## Supporting Information Available

Experimental methods, intensity measurements showing the experimental measurements relative to the saturation regime, measurements on total ion yield, other calculated pulse parameters, and scheme of hypothesized enhancement (PDF)
Transparent Peer Review report available (PDF)
