# Supplementary material for: Determining Key Factors for the Open-Loop Control of Molecular Fragmentation Using Shaped Strong Fields
Source: J Phys Chem Lett. 2024 Dec 12;15(51):12464–9. doi: 10.1021/acs.jpclett.4c02889 (PMC11684007; doi:10.1021/acs.jpclett.4c02889)
Supplement: Supplementary file 1 — jz4c02889_si_001.pdf [file jz4c02889_si_001.pdf]

Supporting Information: Determining Key Factors for the Open-Loop Control of Molecular Fragmentation Using Shaped Strong Fields

Jacob Stamm,† Sung Kwon,† and Marcos Dantus*,†,‡,¶

†*Department of Chemistry, Michigan State University, 48824 East Lansing, MI, United States*

‡*Department of Physics and Astronomy, Michigan State University, 48824 East Lansing, MI, United States*

¶*Department of Electric and Computer Engineering, Michigan State University, 48824 East Lansing, MI, United States*

E-mail: dantus@chemistry.msu.edu

Table of Contents

1 Experimental Methods **S-2**

2 Intensity Relative to Saturation **S-2**

3 Total Ion Yield Measurements **S-3**

4 Other Calculated Pulse Parameters **S-4**

5 Hypothesized Enhancement Scheme **S-5**

References **S-7**

1 Experimental Methods

The femtosecond pulses used in this experiment were generated from an Astrella (Coherent) Ti:Sapphire laser generating 30 fs pulses with a ∼795 nm central wavelength at 1kHz. Pulses had all orders of phase distortion compensated with a pulse shaper (MIIPS HD, Biophotonics Solutions Inc.) containing a spatial light modulator (Hamamatsu, 640 x 800) being controlled by the MIIPS software.[S1] Pulses had an intensity of 2.7×10^{14} W cm^{-2} when focused into the time-of-flight (TOF) chamber by a 200 mm lens and were polarized parallel to the TOF axis. The TOF chamber has a base pressure of 5×10^{-8} Torr and experiments were performed at 1×10^{-5} Torr by leaking triethylamine through a needle valve. Positively charged fragments generated in the interaction region between the focused femtosecond laser and the triethylamine (Mallinckrodt) vapor were accelerated to a field-free region by a +2168 V repeller and +1080 V extractor separated by 1 cm. Ions were detected by a microchannel plate detector (Photonis) at the end of the field-free region and coupled to a oscilloscope (LeCroy WaveRunner 610Zi, 1 GHz) for digitization.

Binary phase masks with 80-bits (80 equally spaced intervals across the spectrum that take either a 0 or π phase value) were chosen for the parameterization of the space due to the mean temporal widths of the resulting pulses. The available pulse duration of a pulse is inversely related to the number of bits in the frequency domain, thus, 80-bit binary phases can produce pulses with structure up to ±2 ps. This, in combination with being divisible by the 800 pixels in the pulse shaper, allows for sufficiently complex pulses to be produced. A smaller parameterization such as 16-bit binary phases would only produce appreciable intensity within ±400 fs and the two-pulse mechanism discussed in the manuscript would be missed. For each phase mask that was tested, the mass spectrum of triethylamine was averaged for 5000 laser shots to minimize the impact of noise. All pulses were checked to be unique within each dataset. The pump-probe data in Figure 4 was obtained by applying a V-shaped spectral phase function and scanning the slope of the linear phase components to produce the pump-probe delay.[S2] Note that as the two linear portions of the phase increases, the pulse shaper adds more wrapping (phases get shifted by integer multiples of 2π until they lie within the range $[-\pi,\pi]$). The effect of significant wrapping is decreased intensity which causes a decrease in ion yield for large delays when using a pulse shaper to do pump probe. Such an effect can be seen in the bottom panels of Figure 4.

2 Intensity Relative to Saturation

Performing control experiments within the saturation regime may lead to missed control mechanisms. To ensure that the data in the manuscript was outside this range, a set of 3400 BPS masks covering the full I_{SHG} range (minimum I_{SHG} to transform-limited) was generated and total ion signal tracked. This is shown in Figure S1 which shows that the data taken in the manuscript (red box) lies before saturation of the focal volume.

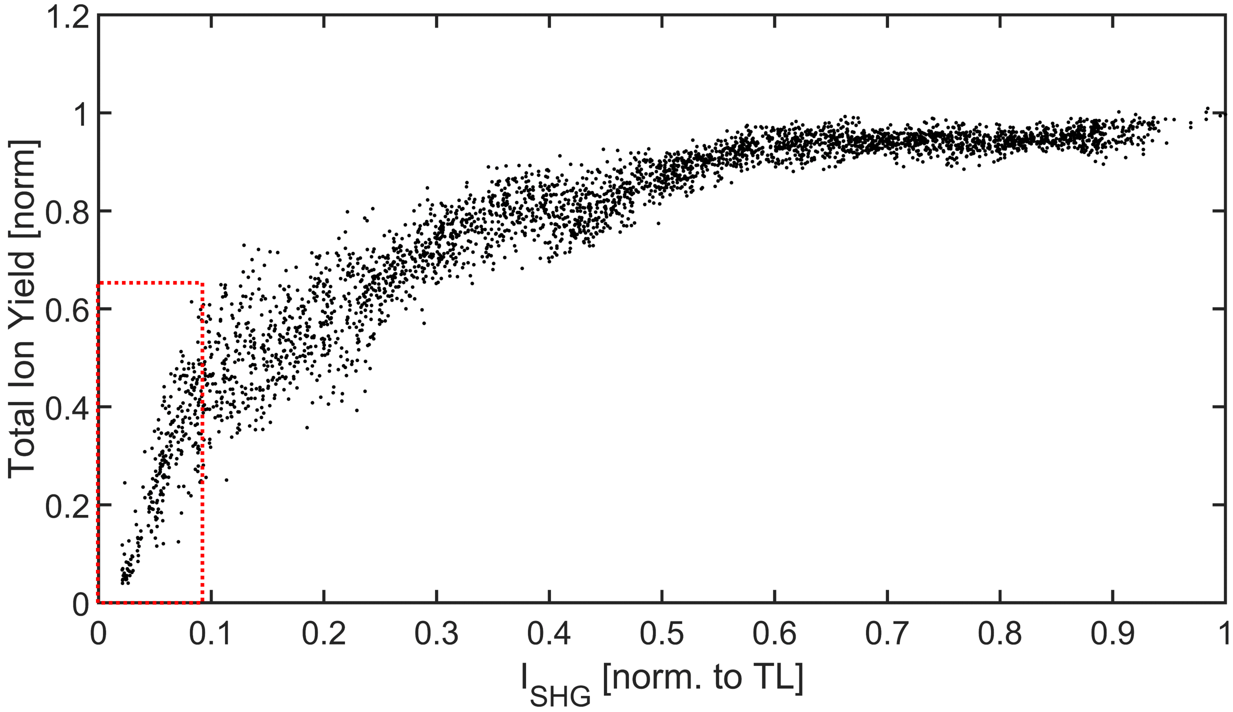

Figure S1: Total ion yield vs normalized integrated second harmonic intensity (I_{SHG}) following the irradiation of triethylamine with 3400 80-bit binary shaped pulses. The set of 3400 masks in this dataset is distinct from the 3200 in the main manuscript. As opposed to the data in the manuscript, these BPS phases were chosen to cover the full I_{SHG} range (minimum I_{SHG} to transform-limited). The red box indicates the regime where the data in the manuscript was taken.

3 Total Ion Yield Measurements

Previous work has shown that the integral of the second harmonic generation spectrum (I_{SHG}) generated by a given shaped femtosecond laser pulse is one of the key parameters that can predict the resulting ion yield. Specifically when fragmenting polyatomic molecules, it has been shown that I_{SHG} is linearly proportional to the total integrated ion signal (I_{MS}).[S3,S4] The data in Figure 3 in the main manuscript involves normalizing the m/z 86 yield to this total ion yield and plotting as a function of I_{SHG}. Figure S2 shows this linearity between I_{SHG} and I_{MS} for BPS pulses. The inset shows that the average 4th harmonic power spectrum in the more-than-expected group is redshifted relative to the less-than-expected group. This indicates that absorption to the Rydberg state of neutral triethylamine aids in further ionization.

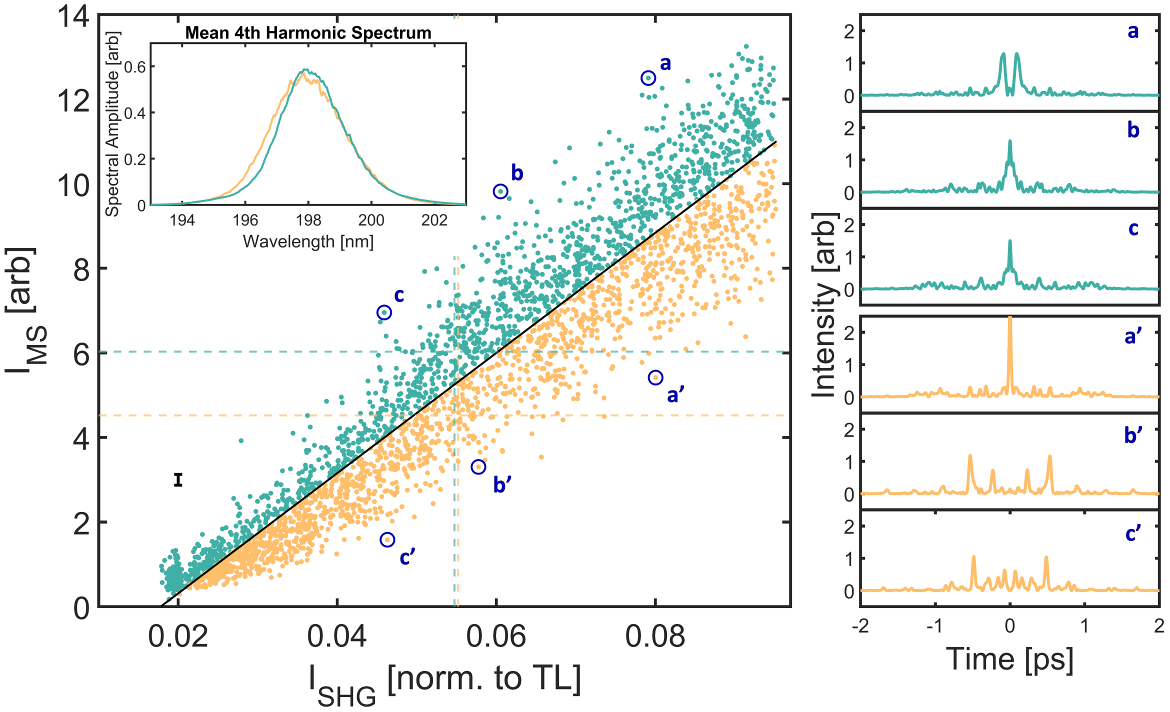

Figure S2: Total ion yield (I_{MS}) vs integrated second harmonic intensity (I_{SHG}) following the irradiation of triethylamine with 3200 80-bit binary shaped pulses. The linear fit to the data is represented as a black line and phases above this line are colored turquoise while phases below this line are colored tan. The average I_{MS} and I_{SHG} values for both of these groups are shown as dashed lines. The black line in the bottom left of the scatter plot indicates the $\pm 1\sigma$ noise level, which was consistent across data points of different total ion signal. In the inset, the mean 4th harmonic power spectra for BPS masks producing more total ion signal than expected by their I_{SHG} are shown in turquoise while those making less than I_{SHG} predicts are shown in tan. The temporal profiles of 6 selected masks (blue circles) are plotted in the right panels.

4 Other Calculated Pulse Parameters

In the main manuscript, it was mentioned that many pulse parameters were considered when attempting to explain the variance in the normalized m/z 86 yield for binary masks of similar I_{SHG} values. Some of the other calculated pulse parameters apart from the PACFs are shown in Figure S3. Some of these parameters reflect the same intrinsic two-pulse interaction mechanism as discussed in the main manuscript. For example, the group producing more-than-expected m/z 86 (turquoise) has a larger mean temporal width (panel d), which is commensurate with biasing towards well-separated pulses on the order of 2 ps.

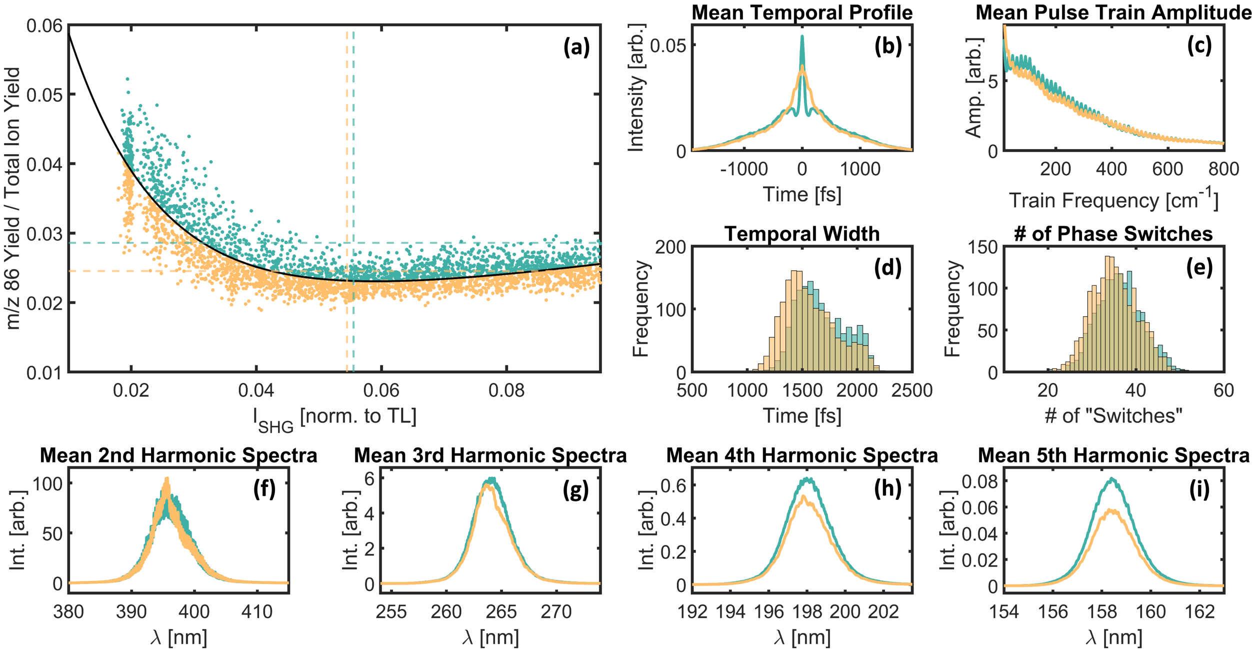

Figure S3: Calculated mean pulse parameters for the group of binary phase masks generating more-than-expected normalized m/z 86 yield (turquoise group) and less-than-expected normalized m/z 86 yield (tan group). Pulse parameters were calculated for each mask individually and subsequently averaged within each group. (a) Normalized m/z 86 yield vs integrated second harmonic power. The functional fit of this data determines the two groups whose pulse parameters are averaged. (b) Mean temporal intensity profiles of the two groups. (c) Mean Fourier transforms of the temporal intensity profiles. (d) Histogram of the mean temporal widths calculated via a weighted average with the temporal intensity profile. (e) Histogram of the number of "switches" (0 to π or π to 0 transitions) within the spectral phase mask for the two groups. (f) Mean 2nd harmonic spectra. (g) Mean 3rd harmonic spectra. (h) Mean 4th harmonic spectra. (i) Mean 5th harmonic spectra.

5 Hypothesized Enhancement Scheme

The enhancement of m/z 86 production when using BPS pulses with a 2-pulse structure was hypothesized in the main manuscript to involve the dynamics of Rydberg states in the neutral triethylamine molecule. It's important to point out that this isn't the only mechanism of m/z 86 production, just the mechanism that explains the enhancement. Figure S4 illustrates the proposed mechanism of m/z 86 production when using a single strong pulse (blue arrows) and when using optimally spaced pulse pairs (orange arrows). Note that the relative contributions to the m/z 86 signal from each of these pathways depend heavily on the intensity used for the pulse(s).

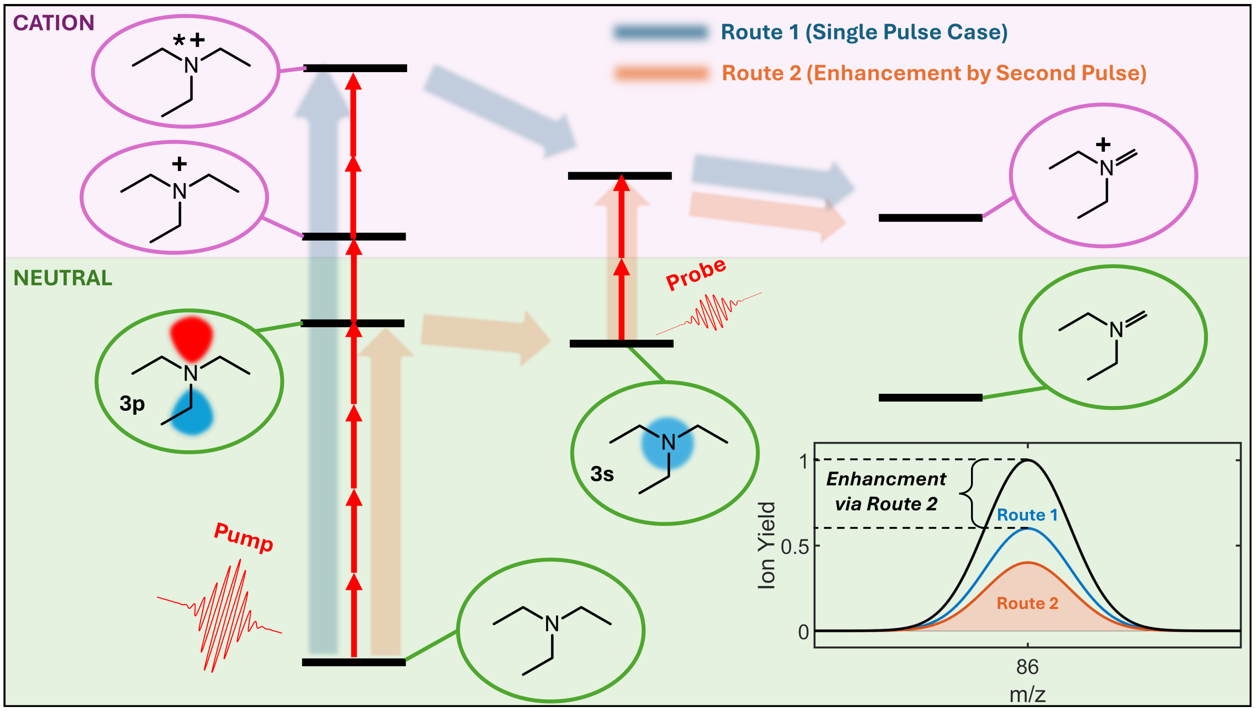

Figure S4: Two proposed pathways of m/z 86 formation from triethylamine. The blue arrows correspond to the m/z 86 formation mechanism when using a single strong pulse. This pathway involves an excited state of the triethylamine cation due to intensity-dependence results showing molecular ion formation prior to m/z 86 formation. The orange arrows correspond to the m/z 86 formation pathway responsible for the controlled enhancement when using shaped pulses, specifically when the shaped pulses approximate peaks separated by 2 ps. Based on the observed time-dependence in Figure 4 of the manuscript, the second pulse most likely ionizes the 3s Rydberg state to a repulsive state of the cation, leading to the formation of m/z 86. Note that the relative energies of the different states and the number of photons required to reach them are estimated for illustration purposes and should not be considered quantitatively. The inset illustrates m/z 86 yield from the two routes (blue and orange) and their combination to form the enhancement seen in the main manuscript (black).

References

(S1) Coello, Y.; Lozovoy, V. V.; Gunaratne, T. C.; Xu, B.; Borukhovich, I.; Tseng, C.-h.; Weinacht, T.; Dantus, M. Interference without an interferometer: a different approach to measuring, compressing, and shaping ultrashort laser pulses. *J. Opt. Soc. Am. B* **2008**, *25*, A140–A150.

(S2) Vogt, G.; Nuernberger, P.; Selle, R.; Dimler, F.; Brixner, T.; Gerber, G. Analysis of femtosecond quantum control mechanisms with colored double pulses. *Phys. Rev. A* **2006**, *74*, 033413.

(S3) Brixner, T.; Kiefer, B.; Gerber, G. Problem complexity in femtosecond quantum control. *Chem. Phys.* **2001**, *267*, 241–246.

(S4) Lozovoy, V. V.; Zhu, X.; Gunaratne, T. C.; Harris, D. A.; Shane, J. C.; Dantus, M. Control of molecular fragmentation using shaped femtosecond pulses. *J. Phys. Chem. A.* **2008**, *112*, 3789–3812.
