# Supplementary material for: Determining Key Factors for the Open-Loop Control of Molecular Fragmentation Using Shaped Strong Fields
Source: J Phys Chem Lett. 2024 Dec 12;15(51):12464–9. doi: 10.1021/acs.jpclett.4c02889 (PMC11684007; doi:10.1021/acs.jpclett.4c02889)
Supplement: Supplementary file 2 — jz4c02889_si_002.pdf [file jz4c02889_si_002.pdf]

jz-2024-028897.R1

Name: Peer Review Information for "Determining Key Factors for the Open-Loop Control of Molecular Fragmentation Using Shaped Strong Fields"

First Round of Reviewer Comments

Reviewer: 1

Comments to the Author

Interesting study by the Dantus group on how pulse shapes affect the yield of methyl loss. There are two things that would improve the legibility of the manuscript. At least a little description of the experiment and for Fig 3 describe in the caption what is a-c and a'-c'. On the scientific side - I am not sure this is making much sense to publish without a hypothesis. All the changes are only resulting in a modification of the distribution of the internal energy in the parent ion. No bond rupture takes place in the neutral so in essence it doesnt matter much how the ground state ion is prepared. The chemistry takes place in the acceleration region on the my-ms time scale. Unless the authors have indications to show that this is not the case its all ion kinetics. Also i wonder how any conclusions can be draw based on a total ion count. To the best of my knowledge that number is pretty arbitrary from one experiment to another.

Reviewer: 2

Comments to the Author

Major advance: The authors demonstrate effective enhancement or depletion of the lowest-energy bond cleavage pathway in triethylamine through open-loop pulse shaping parameter scanning using binary phase shaping. They present a convincing mechanism that relies on pump-probe type excitation involving a 4-photon transition to a Rydberg state and subsequent absorption of a 5th photon after 2 ps to induce methyl loss.

Immediate significance: The main significance is the demonstration that open-loop parameter scanning under very constrained pulse shaping conditions can achieve comparable levels of molecular fragmentation control to closed-loop optimal control experiments performed over the past two decades. However, the authors' finding that the mechanism of fragmentation enhancement is essentially pump-probe control seems to weaken the case for the open-loop pulse

shaping approach since pump-probe measurements can easily be performed without pulse shaping equipment. If the authors can clearly justify why their pulse shaping approach has advantages over pump-probe approaches, then the manuscript will be suitable for publication in J. Phys. Chem. Lett.

Technical suggestions:

1. The authors propose binary phase shaping to reduce the size of the parameter space needed for open-loop searching, but do not provide sufficient motivation for this choice over alternatives such as sinusoidal or polynomial expansions of the spectral phase (e.g. as used in these papers: https://doi.org/10.1063/1.1826011 https://doi.org/10.1063/1.4824153) For instance, the phase shown in Figure 1 looks like it could be parameterized as a linear combination of a second and fourth-order polynomial expansion. What is the advantage of using binary phase shaping as compared to such other parameterizations?

2. In Figure 2, axis labels for the ordinate axis in each mass spectrum are missing and the numbers on these axes are confusing (i.e., it appears that "1" on the axis in panels b and c corresponds different absolute yields even after accounting for the scaling down of the molecular ion). If the absolute values on the ordinate axes of the mass spectra have no meaning, I would recommend removing them and just specifying arbitrary units for ion signal.

3. The caption in Figure S1 states that 3400 binary shaped pulses were used, but the main text states 3200. I assume that 3200 is the correct value, but the numbers should be consistent.

4. The I_SHG values reported in Figures 3 and S2 compared S1 are in different units, which makes direct assessment of the SHG yield/peak intensity in Figure 3 difficult. Could these values be expressed in the same units in all of the figures? The units of relative SHG compared to the TL pulse would be clearest, as in Figure S1.

5. On p. 4, the authors state that differences in multiple pulse parameters between the high- and low-m/z 86 yield pulses with similar I_SHG values were calculated, but do not present the results of these calculations except for the autocorrelation metric in Figure 4. It would be helpful to present the results for the parameters that didn't explain the different m/z 86 yields in the SI to support the statement "No explanation for the 2x difference in normalized m/z 86 ion yield for masks of similar I_SHG values were found".

6. The mathematical definition of the PACF used in Figure 4 is not presented in the text or SI. Although I have some qualitative idea of what this function represents, its formula and method for calculation should be provided in the SI. In particular, it is unclear in Figure 4 whether the Autocorrelation and Difference values reported in on the ordinate axes of the top panel represent ion yields or the laser field strength (I suspect the former, but am not certain).

7. The argument that the control achieved with the high-m/z 86 yield binary phase pulses arises from a pump-probe like mechanism is convincing but raises the question of why perform open loop pulse shaping if the same fragmentation yield can be obtained with a pump-probe pair? Do the best shaped pulses outperform the pump-probe pulse constructed in Figure 4?

Author's Response to Peer Review Comments:

RESPONSES TO COMMENTS BY THE REVIEWERS
In addition, a redlined version of the revised paper has been uploaded.

Comment by Reviewer #1:

Interesting study by the Dantus group on how pulse shapes affect the yield of methyl loss. There are two things that would improve the legibility of the manuscript. At least a little description of the experiment and for Fig 3 describe in the caption what is a-c and a'-c'.

Our response:

We thank the reviewer for these comments. Based on the above points, we have made the following changes to the manuscript:

At the start of the paragraph that begins with "The mass spectrum of triethylamine…" we added the following experimental description:

"Details of the experimental methodology are presented in the Experimental Methods section of the Supporting Information. Briefly, 795-nm, 35-fs laser pulses were shaped with a pulse shaper and focused into a time-of-flight mass spectrometer. Triethylamine vapor was leaked into the mass spectrometer through a needle valve and its resulting mass spectrum following ionization by the laser was recorded for multiple series of binary phase-shaped pulses."

Additionally, we have clarified what the a-c and a'-c' pulses are in the caption of Figure 3 by modifying the final sentence to read:

"The temporal profiles of three selected pairs of masks (a/a', b/b', and c/c', blue circles), each with similar I_{SHG} values but large differences in m/z 86 yield, are plotted in the right panels."

Comment by Reviewer #1:

On the scientific side - I am not sure this is making much sense to publish without a hypothesis. All the changes are only resulting in a modification of the distribution of the internal energy in the parent ion. No bond rupture takes place in the neutral so in essence it doesnt matter much how the ground state ion is prepared. The chemistry takes place in the acceleration region on the my-ms time scale. Unless the authors have indications to show that this is not the case its all ion kinetics. Also i wonder how any conclusions can be draw based on a total ion count. To the best of my knowledge that number is pretty arbitrary from one experiment to another.

Our response:

The reviewer brings up several points. First, we agree that a hypothesis would benefit the manuscript. To address this, we have changed the last sentence of the introduction that read "Such explanatory variables and guiding principles…" to:

"It is expected that using an open-loop search with BPS parameterization will enable the extraction of critical pulse parameters necessary for controlling molecular fragmentation. This approach may reveal new control mechanisms without a priori knowledge of the system under study."

In our manuscript, we propose that the control over triethylamine's fragmentation, achieved through a two-pulse mechanism identified using an open loop search, involves a Rydberg state in the neutral molecule. This conclusion is based on the optimal time delay of 2 ps between the pulses. Therefore, we do not agree that the results in the manuscript are attributable to the internal energy of the parent ion.

While internal energy is a common confounding variable in coherent control experiments, we address this issue by plotting the ion yield as a function of the second-harmonic generation intensity of the pulses used (I_{SHG}). Pulses with

the same I_{SHG} have the same peak intensity, regardless of their temporal structure. Furthermore, given that the pump-probe results in Figure 4 point towards control over the m/z 86 yield in the 1-3 ps regime, we disagree that the relevant chemistry is happening in the field-free region of the mass spectrometer. While some fragmentation could occur during the long (µs-ms) timescale, the degree of control over m/z 86 shown here is independent of such chemistry given the optimal ~2 ps pump-probe delay found.

Finally, we agree that total ion count can be a difficult quantity to compare between experiments and certain measures need to be taken to ensure reproducibility. This is why we report the m/z 86 yield normalized to the total ion signal, a ratio that inherently accounts for many experimental differences. We emphasize that no signal in the main manuscript is reported as total ion count. In response to this comment, as well as comments by Reviewer #2, we have updated the I_{SHG} axis in all experimental plots to be relative to the integrated second harmonic power spectrum of the transform-limited pulse (see pages 5 and 6 of this document).

Comment by Reviewer #2:

Major advance: The authors demonstrate effective enhancement or depletion of the lowest-energy bond cleavage pathway in triethylamine through open-loop pulse shaping parameter scanning using binary phase shaping. They present a convincing mechanism that relies on pump-probe type excitation involving a 4-photon transition to a Rydberg state and subsequent absorption of a 5th photon after 2 ps to induce methyl loss.

Immediate significance: The main significance is the demonstration that open-loop parameter scanning under very constrained pulse shaping conditions can achieve comparable levels of molecular fragmentation control to closed-loop optimal control experiments performed over the past two decades. However, the authors' finding that the mechanism of fragmentation enhancement is essentially pump-probe control seems to weaken the case for the open-loop pulse shaping approach since pump-probe measurements can easily be performed without pulse shaping equipment. If the authors can clearly justify why their pulse shaping approach has advantages over pump-probe approaches, then the manuscript will be suitable for publication in J. Phys. Chem. Lett.

Our response:

We thank the reviewer for the constructive comments. Regarding the use of open-loop phase shaping instead of typical pump-probe measurements: The goal of the manuscript is to illustrate a generalized recipe to control the fragmentation of polyatomic molecules using open-loop binary phase shaping without a priori knowledge. This scheme involves a few steps.

- Exciting the system with a series of different binary-phase-shaped (BPS) pulses and recording the control over the desired outcome.
- Accounting for intensity effects by plotting the control objective vs I_{SHG}.
- Calculating pulse properties such as the temporal partial autocorrelation function and others shown in Fig. S3 for each BPS pulse.
- Determining which pulse properties account for the variance in the control target across masks of similar I_{SHG}.

In the specific system under study in the manuscript, the pulse parameter elicited control over triethylamine was the "pump-probe" structure of the temporal profiles. The reviewer is correct that a pump-probe scan would also find such an enhancement of methyl loss in this specific system (Figure 4, bottom left panel shows the result of a pump-probe scan). However, not all systems may require a pump-probe structure to elicit control over a given fragmentation channel. It's been seen in the literature that other phases (such as chirp, sinusoidal, etc.) may be needed to control a given quantum mechanical process. The open-loop BPS methodology presented here would be able to find such solutions given its ability to approximate any spectral phase function (as shown in Figure 1). We would also like to emphasize that spectral phases that produce pump-probe structures are included within the BPS parameterization, so BPS allows for the discovery of pump-probe optimal solutions (as shown in the manuscript), as well as different solutions for other quantum systems.

We agree that this argument should be explicitly stated in the manuscript. We have added the following sentence to the end of the Introduction section to emphasize that this is a general approach to controlling molecular fragmentation:

"It is expected that using an open-loop search with BPS parameterization will enable the extraction of critical pulse parameters necessary for controlling molecular fragmentation. This approach may reveal new control mechanisms without a priori knowledge of the system under study."

To compare our method with pump-probe results, we have added a red vertical line to Figure 5 indicating the maximum normalized m/z 86 yield obtained using a V-shaped spectral phase to generate pump-probe pulse pair. We have also included an inset showing the temporal profiles for the best (blue) and worst (orange) 80-bit shaped pulses.

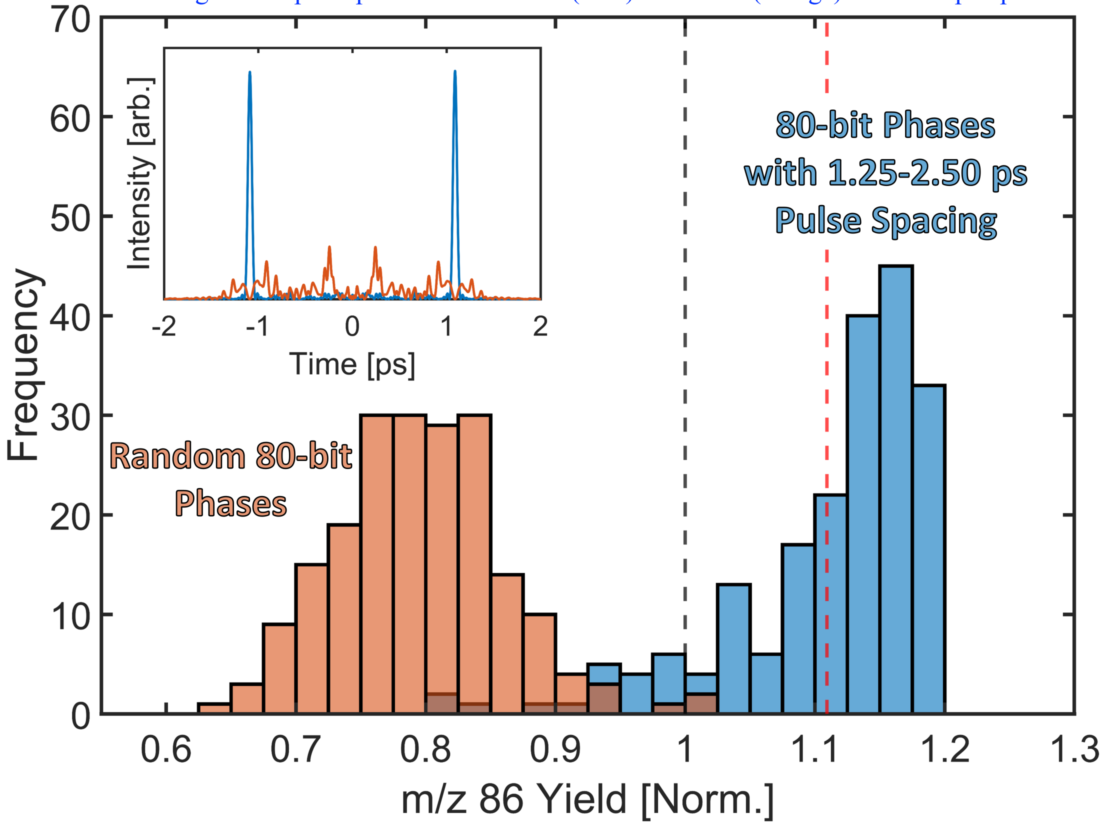

To reflect this change, we have updated the caption of Figure 5 to read:

"Figure 5: Histogram of the normalized m/z 86 yield for 200 randomly generated 80-bit binary phases (orange) and 200 80-bit binary phases with pulse structures separated by 1.25 to 2.5 ps (blue). Both groups have the same integrated second harmonic intensity and have been normalized to the m/z 86 yield for a single transform-limited pulse (black dotted line). The maximum normalized m/z 86 yield obtained via pump-probe measurements using a V-shaped spectral phase function is shown as a red dotted line. The inset shows the temporal profiles for the best mask in the BPS spaced pulses group (blue) and worst mask in the random BPS group (orange)."

To discuss this change in the manuscript, we have changed the sentence that reads "Additionally, the pulses chosen with ps pulse structure…" to:

"Additionally, the selected shaped pulses with a double-pulse structure and picosecond spacing outperform both transform-limited pulses (black dashed line) and pump-probe results (red dashed line). This demonstrates an extra enhancement of the BPS-shaped pulses, which may be attributed to pulse replication effects caused by the 80-bit pixelation of the phases."

Comment by Reviewer #2:

The authors propose binary phase shaping to reduce the size of the parameter space needed for open-loop searching, but do not provide sufficient motivation for this choice over alternatives such as sinusoidal or polynomial expansions of the spectral phase (e.g. as used in these papers: doi.org/10.1063/1.1826011, doi.org/10.1063/1.4824153) For instance, the phase shown in Figure 1 looks like it could be parameterized as a linear combination of a second and fourth-order polynomial expansion. What is the advantage of using binary phase shaping as compared to such other parameterizations?

Our response:

We agree that the choice of phase space parameterization must be well-justified. While polynomial phases and sinusoidal phases can be used in an open-loop manner with the same approach used in the manuscript, we believe that BPS is the most natural and economical for controlling such processes. Binary phase functions are especially well-suited for controlling quantum excitation pathways through constructive and destructive interference. Specifically, a π-phase difference between two pathways results in destructive interference, while a 0-phase difference leads to constructive interference. Furthermore, binary phases are uniquely reproducible because the parameters (0 and π) can be easily calibrated through interference. The other parameterizations the reviewer mentions do have their upsides: sinusoidal phase control is particularly effective for generating pulse trains, and polynomial expansions are effective for pulse compression. These other parameterizations can be applied in an open-loop manner using the same methodology outlined in the manuscript but depending on the optimal phase mask may require a large number of parameters.

To explain to the reader these alternative parameterization schemes and why BPS was specifically chosen, the following was added to the end of the second to last paragraph of the Introduction:

"Note that other parameterizations besides BPS are possible for open-loop searches and could be applied in an open-loop manner using the same methodology outlined here. Some of these alternatives include sinusoidal phase control, which is particularly effective for generating pulse trains,[16-18] and polynomial expansions, which are practical for pulse compression as pulse broadening is primarily influenced by low-order components (2nd to 5th).[19] However, representing any arbitrary phase function using these parameterizations would necessitate a large sum of sinusoidal functions (as in a sinusoidal transform) or high-order polynomials (as in a Taylor expansion). Depending on the optimal phase function, a large number of parameters may be required in these expansions. Here, BPS was chosen due to its simplicity and reproducibility[20] (only 0 and π phase values need to be calibrated), as well as its tailoring towards controlling quantum excitation pathways through constructive (0-phase difference) or destructive (π-phase difference) pathway interference.[21]"

Comment by Reviewer #2:

In Figure 2, axis labels for the ordinate axis in each mass spectrum are missing and the numbers on these axes are confusing (i.e., it appears that "1" on the axis in panels b and c corresponds different absolute yields even after accounting for the scaling down of the molecular ion). If the absolute values on the ordinate axes of the mass spectra have no meaning, I would recommend removing them and just specifying arbitrary units for ion signal.

Our response:

We agree that this may be confusing. The proper axis label (Normalized Ion Yield) has been added to the ordinate axis of Figure 2. The numbers on this axis are important for the interpretation and comparison between mass spectra. These numbers are obtained by dividing the raw ion yields by the total ion signal. This allows for better comparison between the spectra and explains why the same y-value seems to correspond to different absolute yields.

The following has been added after the first sentence in the caption of Figure 2:

"All spectra have been normalized to the total integrated ion signal."

Comment by Reviewer #2:

The caption in Figure S1 states that 3400 binary shaped pulses were used, but the main text states 3200. I assume that 3200 is the correct value, but the numbers should be consistent.

Our response:

For the data in the main text, the correct value for the number of masks tested is 3200. In contrast, the data in Figure S1 is based on a different set of 3400 masks that cover the entire SHG range. Since the two numbers are close, the following clarification has been added to the caption of Figure S1 before the sentence "As opposed to the data in the manuscript…":

"The set of 3400 masks in this dataset is distinct from the 3200 in the main manuscript."

Comment by Reviewer #2:

The I_SHG values reported in Figures 3 and S2 compared S1 are in different units, which makes direct assessment of the SHG yield/peak intensity in Figure 3 difficult. Could these values be expressed in the same units in all of the figures? The units of relative SHG compared to the TL pulse would be clearest, as in Figure S1.

Our response:

We agree with the reviewer's suggestion and have made all I_{SHG} values in the main text relative to transform-limited. Below are the updated Figure 3 and Figure S2.

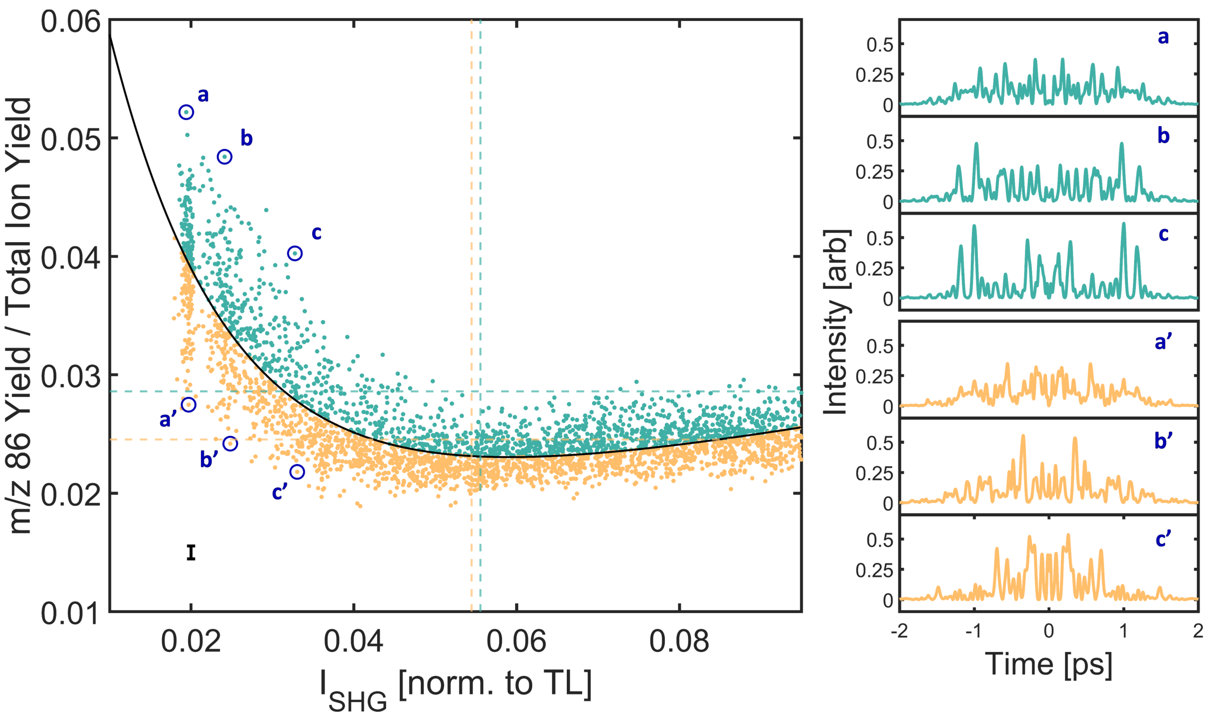

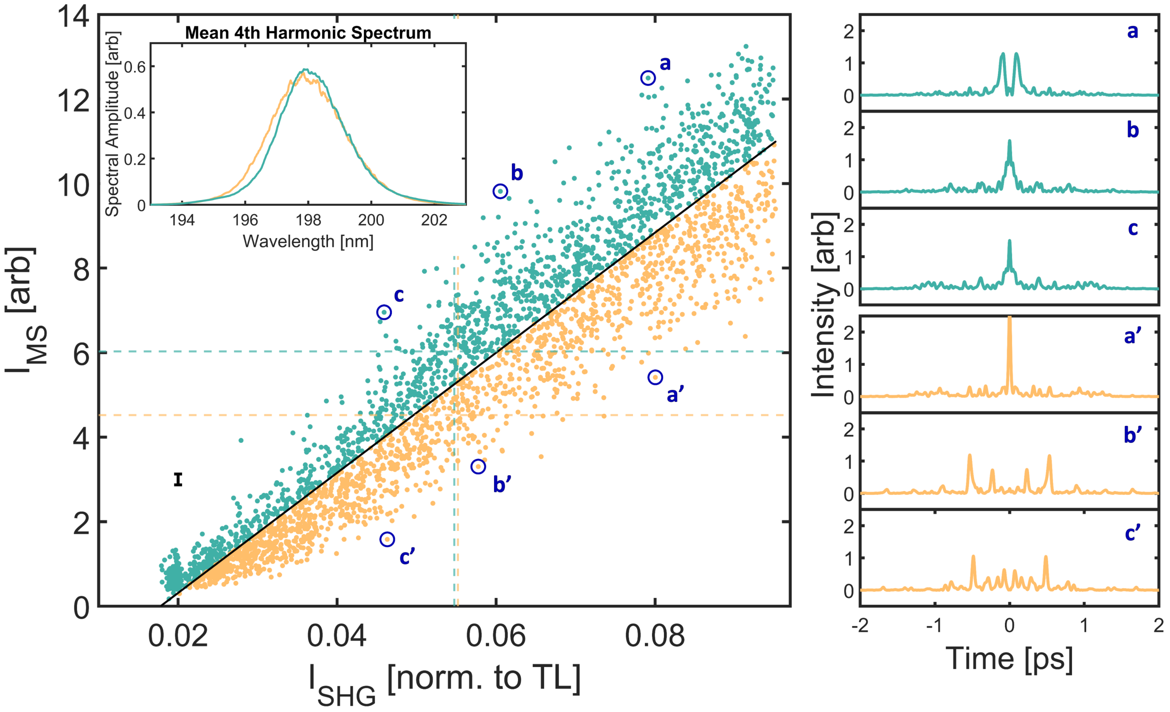

Comment by Reviewer #2:

On p. 4, the authors state that differences in multiple pulse parameters between the high- and low-m/z 86 yield pulses with similar I_SHG values were calculated, but do not present the results of these calculations except for the autocorrelation metric in Figure 4. It would be helpful to present the results for the parameters that didn’t explain the different m/z 86 yields in the SI to support the statement “No explanation for the 2x difference in normalized m/z 86 ion yield for masks of similar I_SHG values were found”.

Our response:

We agree with the reviewer’s suggestion as it emphasizes that using this method for other systems may yield a control mechanism involving a different pulse parameter (apart from PACF). Figure S3 has been added to the Supporting Information to show the differences in other parameters between the two groups:

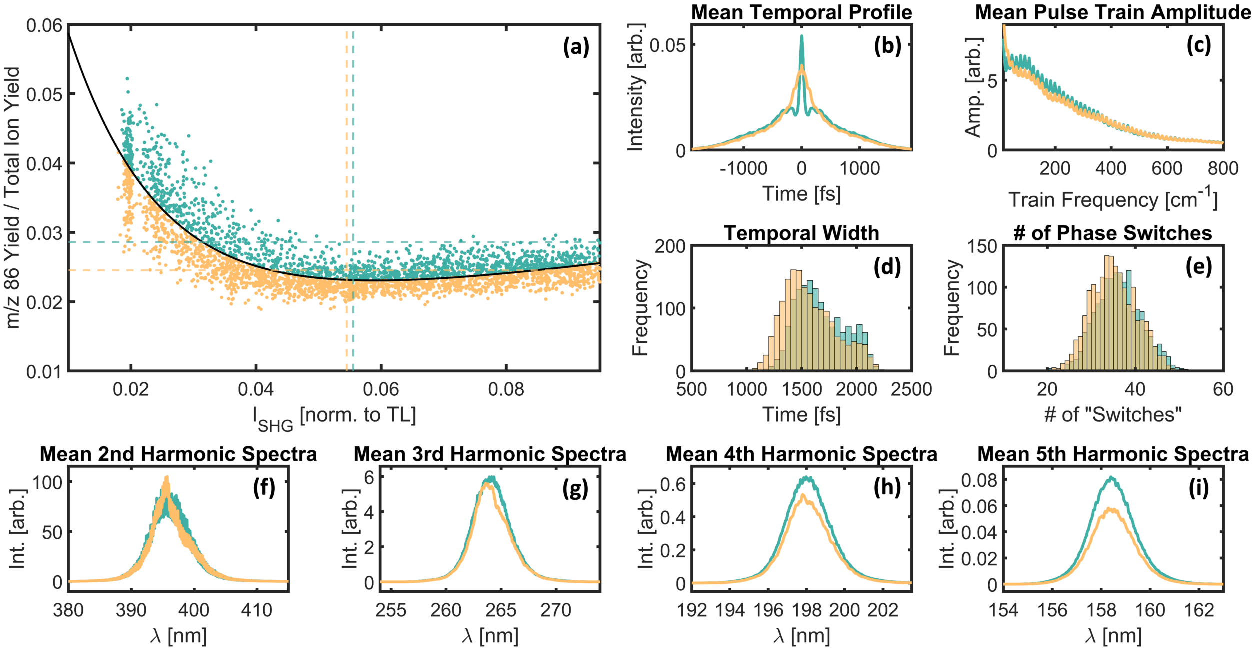

"Figure S3: Calculated mean pulse parameters for the group of binary phase masks generating more-than-expected normalized m/z 86 yield (turquoise group) and less-than-expected normalized m/z 86 yield (tan group). Pulse parameters were calculated for each mask individually and subsequently averaged within each group. (a) Normalized m/z 86 yield vs integrated second harmonic power. The functional fit of this data determines the two groups whose pulse parameters are averaged. (b) Mean temporal intensity profiles of the two groups. (c) Mean Fourier transforms of the temporal intensity profiles. (d) Histogram of the mean temporal widths calculated via a weighted average with the temporal intensity profile. (e) Histogram of the number of "switches" (0 to π or π to 0 transitions) within the spectral phase mask for the two groups. (f) Mean 2nd harmonic spectra. (g) Mean 3rd harmonic spectra. (h) Mean 4th harmonic spectra. (i) Mean 5th harmonic spectra."

Additionally, a new section titled "Other Calculated Pulse Parameters" has been added to the Supporting Information as well as the following text discussing Figure S3:

"In the main manuscript, it was mentioned that many pulse parameters were considered when attempting to explain the variance in the normalized m/z 86 yield for binary masks of similar I_{SHG} values. Some of the other calculated pulse parameters apart from the PACFs are shown in Figure S3. Some of these parameters reflect the same intrinsic two-pulse interaction mechanism as discussed in the main manuscript. For example, the group producing more-than-expected m/z 86 (turquoise) has a larger mean temporal width (panel d), which is commensurate with biasing towards well-separated pulses on the order of 2 ps."

Comment by Reviewer #2:

The mathematical definition of the PACF used in Figure 4 is not presented in the text or SI. Although I have some qualitative idea of what this function represents, its formula and method for calculation should be provided in the SI. In particular, it is unclear in Figure 4 whether the Autocorrelation and Difference values reported in on the ordinate axes of the top panel represent ion yields or the laser field strength (I suspect the former, but am not certain).

Our response:

Given that the PACFs are the key control parameter in this manuscript, we agree that further clarification is needed. The ordinate axis of Figure 4 represents the average autocorrelation of the temporal profiles of BPS masks making more (turquoise) or less (tan) normalized m/z 86 than I_{SHG} predicts.

To clarify, the formula for PACFs has been added to the paragraph where PACFs are introduced in the manuscript. After the sentence reading “These PACFs are simply the correlation of a temporal profile with a delayed copy of itself…” we add:

“This value is proportional to:

$$A(\tau) = \int_{-\infty}^{\infty} I(t)I(t-\tau)\mathrm{d}t$$

Where $I(t)$ is the temporal intensity profile of a given pulse, which is calculated via the Fourier transform:

$$I(t) \propto \left| \int_{-\infty}^{\infty} \sqrt{S(\omega)}\mathrm{e}^{-\mathrm{i}\varphi(\omega)}\mathrm{e}^{\mathrm{i}\omega t}\mathrm{d}\omega \right|^2$$

Where $S(\omega)$ is the pulse spectrum and $\varphi(\omega)$ is the spectral phase.”

Additionally, the ordinate axis derivation has been made clearer by the following sentence to the same paragraph after the sentence reading “The mean PACFs for the two groups…”:

“To calculate these quantities, we first compute the PACF for each temporal profile in both groups (turquoise and tan) as a function of time delay τ. Next, we average the PACFs of all masks in each group, resulting in two mean PACFs, illustrated in turquoise and tan.”

Comment by Reviewer #2:

The argument that the control achieved with the high-m/z 86 yield binary phase pulses arises from a pump-probe like mechanism is convincing but raises the question of why perform open loop pulse shaping if the same fragmentation yield can be obtained with a pump-probe pair? Do the best shaped pulses outperform the pump-probe pulse constructed in Figure 4?

Our response:

The reviewer is correct that a pump-probe scan would equally find such an enhancement of methyl loss in this specific system (Figure 4, bottom left panel). However, not all systems may require a pump-probe structure to elicit control over a given fragmentation channel. Other phase structures may be required to control different systems/processes and the open-loop BPS methodology presented here would be able to find such solutions given its ability to approximate any spectral phase function (as shown in Figure 1). The results in the manuscript lay out a generalized way to determine control mechanisms via calculation of a series of pulse parameters. The BPS parameterization is able to create pump-probe structures while also being able to produce optimal solutions to other quantum systems that may require a different pulse structure. Additionally, the optimal BPS masks shown in the manuscript outperform pump-probe results as shown in the updated Figure 5 (see page 3 of this document).

jz-2024-028897.R2

Name: Peer Review Information for "Determining Key Factors for the Open-Loop Control of Molecular Fragmentation Using Shaped Strong Fields"

Second Round of Reviewer Comments

Reviewer: 2

Comments to the Author

The authors have effectively addressed my comments in the original version. I believe the work is now suitable for publication, subject to a couple of minor suggestions:

- In the new Introduction text on p. 2 comparing the BPS with other parameterizations, the authors indicate the possible large parameter space for sinusoidal or polynomial expansions as detrimental, but corresponding parameter reduction with the BPS scheme isn't explained. I actually find the argument that the BPS can approximate any spectral phase to be a stronger motivation for using it instead of other parameterizations, so it would be nice to see this explicitly stated in the introduction because this would build on the authors' argument that the BPS scheme is "agnostic" to any particular control scheme (like pump-probe or pulse compression) and thus useful to find new control regimes.
- In the discussion of Figure 5 about further enhancement of the m/z 86 yield for the best BPS pulses compared to the pump-probe pair, the authors attribute the enhancement to "pulse replication effects caused by the 80-bit pixelation". A bit more explanation here is warranted: is the "pulse replication" due to phase wrapping and does this result in more than two effective sub-pulses (instead of just two as in the pump-probe)? Is it possible that the 80-bit resolution that results in the 2.2 ps "spikes" was a coincidentally lucky choice of resolution that happened to overlap with a good pump-probe delay for this particular molecule? In my opinion it would strengthen the argument for the BPS open loop approach to have a bit more discussion/emphasis on why the BPS scheme beats the pump-probe.

Reviewer: 1

Comments to the Author

All but one comment taken care of. Please see the attached drawing. That is how m/z 86 arise. Thus, it doesnt reflect any of the dynamics of the neutral. If you have evidence to show that this is not the case I recommend to present it prior to publication.

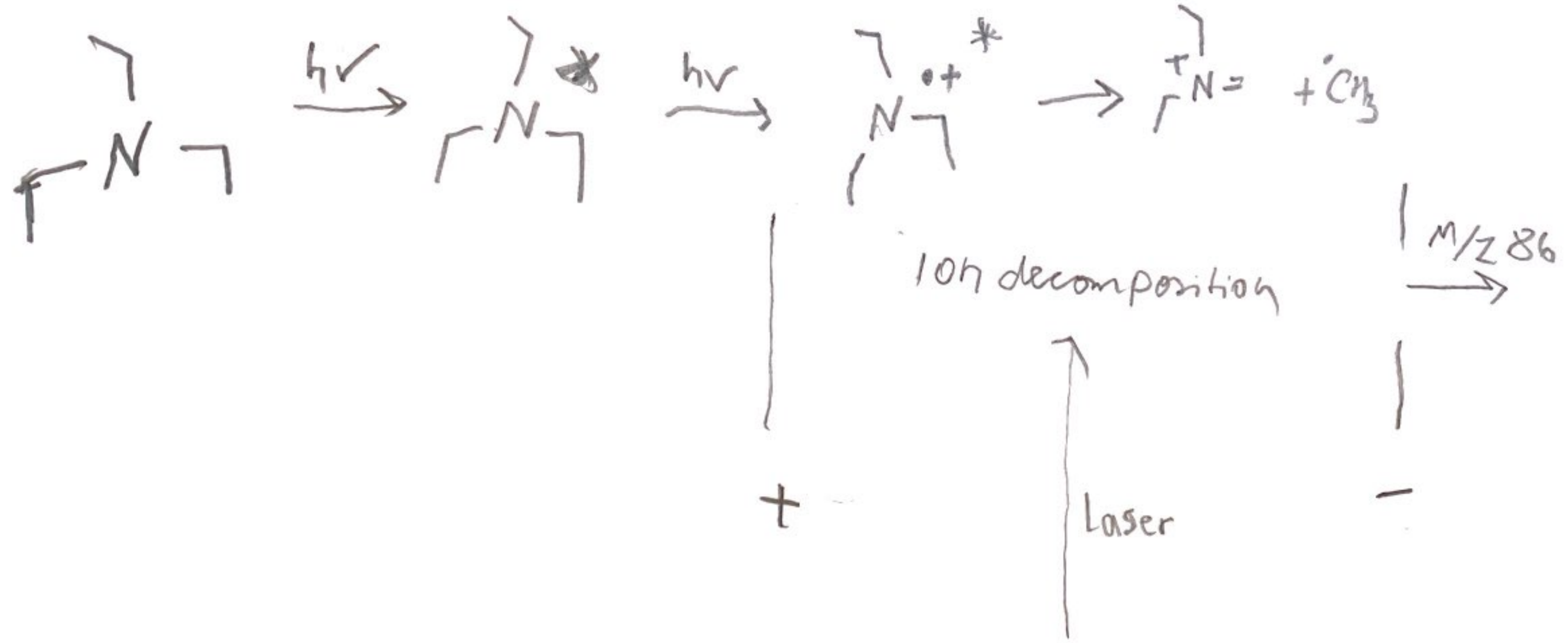
hv
hv
Ion decomposition
m/z 86
Laser
+
−

RESPONSES TO COMMENTS BY THE REVIEWERS

Comment by Reviewer #1:

All but one comment taken care of. Please see the attached drawing. That is how m/z 86 arise. Thus, it doesnt reflect any of the dynamics of the neutral. If you have evidence to show that this is not the case I recommend to present it prior to publication.

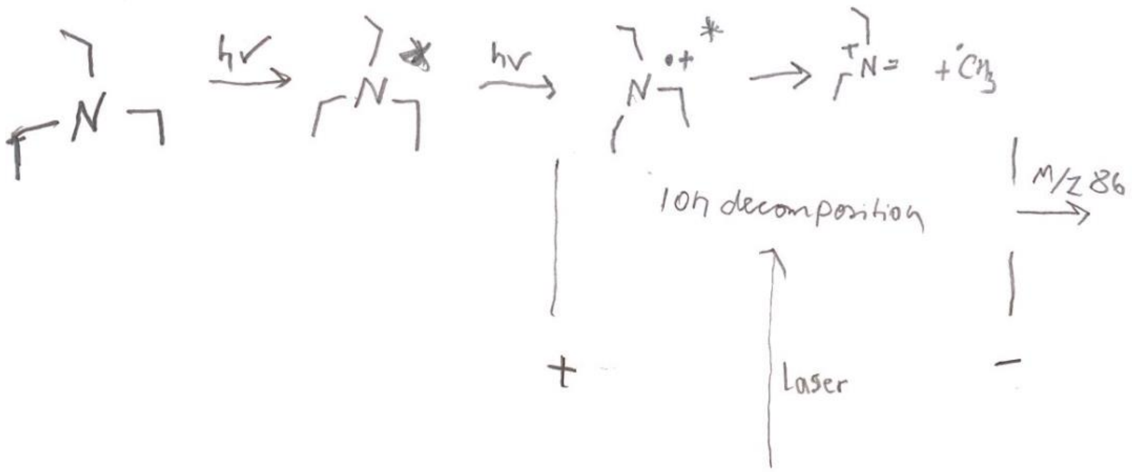

Our response:

The reviewer's scheme correctly suggests that multiphoton ionization of TEA can lead to the formation of m/z 86, a pathway that is indeed observed when ionizing with a single intense short pulse. However, the manuscript focuses on an alternate pathway that depends on a two-pulse structure, responsible for the enhancement observed for certain BPS pulses.

Based on the observed change in m/z 86 yield as a function of the pump-probe delay (Figure 4), with an optimum at 2 ps, we proposed an alternative formation pathway consistent with the observed enhancement that depends on the timing of the second pulse. This pathway, discussed on pages 6 and 7 of the manuscript, involves the population of neutral Rydberg states by the pump pulse, followed by ionization by the probe pulse. It is known that the 3s Rydberg state in similar amines has a ~2 ps lifetime.[1,2] Therefore, the time delay must be long enough to allow relaxation to the 3s Rydberg state but not so long that the state decays before the probe pulse arrives. If the enhancement mechanism involved the cation, we would expect to see an enhancement in other ions, such as m/z 28, 30, and 58. However, this is not observed. To clarify, we have added Figure S4 to the Supporting Information, which illustrates (see below) two pathways for m/z 86 production. The first pathway is the same as the mechanism indicated by the reviewer and is initiated by a single intense pulse (indicated by blue arrows), while the second pathway is triggered by optimally spaced pulse pairs (indicated by orange arrows). Note that the relative contribution to the m/z 86 signal from each pathway depends on the laser pulse intensity. At low intensity, even a single pulse produces only the molecular ion and no m/z 86. At high intensity, the pathway involving the cation becomes more prominent.

[1] Cardoza, J. D.; Rudakov, F. M.; Weber, P. M. Electronic spectroscopy and ultrafast energy relaxation pathways in the lowest rydberg states of trimethylamine. *J. Phys. Chem. A.* **2008**, *112*, 10736–10743.
[2] Gosselin, J. L.; Minitti, M. P.;Rudakov, F. M.; Sølling, T. I.;Weber, P. M. Energy flow and fragmentation dynamics of N, N-dimethylisopropylamine. *J. Phys. Chem. A.* **2006**, *110*, 4251–4255.

The following text was added before Figure S4 in the Supporting Information:

"The enhancement of m/z 86 production when using BPS pulses with a 2-pulse structure was hypothesized in the main manuscript to involve the dynamics of Rydberg states in the neutral triethylamine molecule. It's important to point out that this isn't the only mechanism of m/z 86 production, just the mechanism that explains the enhancement. Figure S4 illustrates the proposed mechanism of m/z 86 production when using a single strong pulse (blue arrows) and when using optimally spaced pulse pairs (orange arrows). Note that the relative contributions to the m/z 86 signal from each of these pathways depend heavily on the intensity used for the pulse(s)."

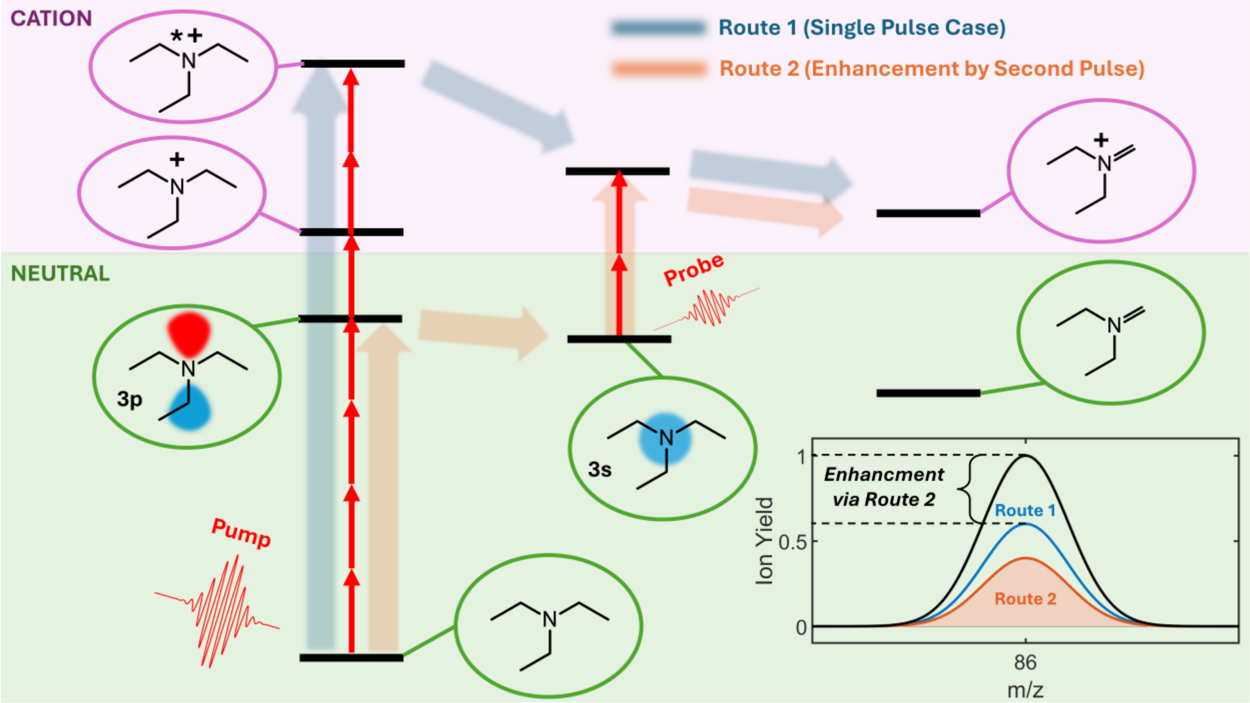

Figure S4: Two proposed pathways of m/z 86 formation from triethylamine. The blue arrows correspond to the m/z 86 formation mechanism when using a single strong pulse. This pathway involves an excited state of the triethylamine cation due to intensity-dependence results showing molecular ion formation prior to m/z 86 formation. The orange arrows correspond to the m/z 86 formation pathway responsible for the controlled enhancement when using shaped pulses, specifically when the shaped pulses approximate peaks separated by 2 ps. Based on the observed time-dependence in Figure 4 of the manuscript, the second pulse most likely ionizes the 3s Rydberg state to a repulsive state of the cation, leading to the formation of m/z 86. Note that the relative energies of the different states and the number of photons required to reach them are estimated for illustration purposes and should not be considered quantitatively. The inset illustrates m/z 86 yield from the two routes (blue and orange) and their combination to form the enhancement seen in the main manuscript (black).

In response to the reviewer's concerns, we have clarified in the manuscript that the Rydberg dynamics discussed specifically refer to the changes in m/z 86 yield induced by two-pulse temporal profile of the shaped pulses and do not represent the only pathway to m/z 86 production. The revised paragraph now reads:

"Given that the optimum pulses involve a two-pulse temporal profile, we propose that the first pulse populates the 3p Rydberg state of the neutral molecule via 4-photon excitation. The 3p Rydberg state then internally converts to the dissociative 3s Rydberg state within a picosecond, when the time-delayed second pulse ionizes the system, populating a repulsive state in the cation that produces m/z 86. The m/z 86 ion signal decreases at longer times as the 3s Rydberg state population decays via α-cleavage, fluorescence, or conversion back to the ground state. This proposed pathway aligns with the pump-probe data shown in Figure 4, which displays a rise and decay consistent with the population and subsequent depopulation of a state generated by the pump pulse. It should be emphasized that this dynamical model explains the enhancement of m/z 86 production caused by the second pulse. The pathway for m/z 86 production

with a single pulse involves direct excitation to a cationic excited state, which also produces m/z 86. This ionic pathway contributes to a background signal that is not observed at low intensities. Thus, the ionic pathway adds a background yield onto which the second-pulse enhancement is superimposed. The two pathways responsible for m/z 86 production are illustrated in Figure S4."

Comment by Reviewer #2:

The authors have effectively addressed my comments in the original version. I believe the work is now suitable for publication, subject to a couple of minor suggestions:

- In the new Introduction text on p. 2 comparing the BPS with other parameterizations, the authors indicate the possible large parameter space for sinusoidal or polynomial expansions as detrimental, but corresponding parameter reduction with the BPS scheme isn't explained. I actually find the argument that the BPS can approximate any spectral phase to be a stronger motivation for using it instead of other parameterizations, so it would be nice to see this explicitly stated in the introduction because this would build on the authors' argument that the BPS scheme is "agnostic" to any particular control scheme (like pump-probe or pulse compression) and thus useful to find new control regimes.

Our response:

The ability of BPS to approximate any spectral phase function is an important point of the paper and justifies its use with an open-loop approach. This is why Figure 1 shows in the Introduction of the paper why BPS is useful. We have changed the sentence referring to this figure in the Introduction section (the sentence beginning with: "Figure 1 shows how a binary"….) to:

"The benefit of BPS is that it can approximate any spectral phase function (Figure 1) while reducing the dimensionality of the phase space that needs to be searched. This retains the capability of uncovering various control mechanisms while allowing for a variable-sized search space (via the number of bits) that may be scaled depending on the number of desired parameters and the system to be optimized."

Comment by Reviewer #2:

- In the discussion of Figure 5 about further enhancement of the m/z 86 yield for the best BPS pulses compared to the pump-probe pair, the authors attribute the enhancement to "pulse replication effects caused by the 80-bit pixelation". A bit more explanation here is warranted: is the "pulse replication" due to phase wrapping and does this result in more than two effective sub-pulses (instead of just two as in the pump-probe)? Is it possible that the 80-bit resolution that results in the 2.2 ps "spikes" was a coincidentally lucky choice of resolution that happened to overlap with a good pump-probe delay for this particular molecule? In my opinion it would strengthen the argument for the BPS open loop approach to have a bit more discussion/emphasis on why the BPS scheme beats the pump-probe.

Our response:

Firstly, the reviewer is correct that the replication effects are caused by the repetitive phase features in the 80-bit BPS masks, and they do indeed lead to satellite pulses in the time domain. These satellites at most are ~10% of the peak intensity of the main pulse(s), as shown below for an 80-bit BPS pulse with a pattern "010101010101…..":

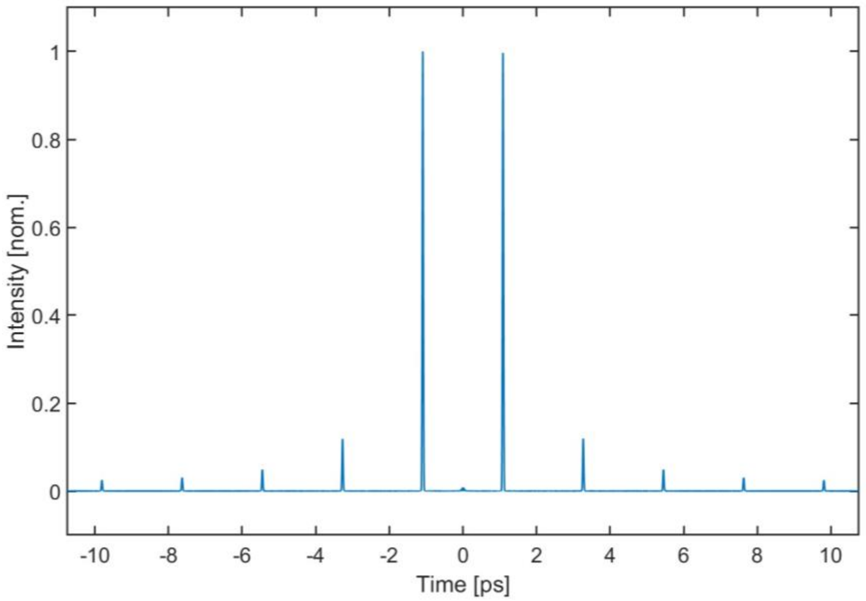

Given that the 80-bit pulses in the manuscript are already quite weak in terms of peak intensity, it would be surprising if these satellites were to have a large effect. However, given that neutral state dynamics are the proposed dynamics mechanism, perhaps these satellites explain the enhancement of BPS pulses relative to pump-probe as they are significantly less likely to ionize the molecule. We have updated the explanation in the text as follows:

"This demonstrates an extra enhancement of the BPS-shaped pulses, which might be attributed to low-intensity satellite pulses that are generated at regular periods from the main pulse(s). These satellites are created due to the repetitive phase features in the 80-bit masks and could contribute to neutral excitation while avoiding ionization due to their low intensity."

Finally, the 80-bit BPS parameterization was chosen because it is large enough to create pulse structures spanning a few picoseconds and because it is divisible by the 800 pixels in our pulse shaper. The "central region" of the temporal intensity profiles goes from -2200 fs to 2200 fs, meaning that pump-probe delays below 4400 fs are possible within an 80-bit BPS parameterization. Molecules that require a pump-probe structure beyond this 4400-fs range would need a larger number of bits (100-bit, 200-bit, etc…) to find such a solution. However, many molecular dynamics measured with pump-probe tend to occur on a faster timescale than that afforded by 80-bit phases, allowing for a more coarse parameterization like 16-bit BPS in such cases.
